# From bench to bedside: cutting-edge applications of base editing and prime editing in precision medicine

**DOI:** 10.1186/s12967-024-05957-3

**Published:** 2024-12-20

**Authors:** Weihui Xu, Shiyao Zhang, Huan Qin, Kai Yao

**Affiliations:** 1 Institute of Visual Neuroscience and Stem Cell Engineering, Wuhan University of Science and Technology, Wuhan, 430065 China; 2 College of Life Sciences and Health, Wuhan University of Science and Technology, Wuhan, 430065 China

**Keywords:** CRISPR–Cas, Genome editing, Base editing, Prime editing, Gene therapy delivery, Clinical treatment, Gene and cell therapy

## Abstract

CRISPR-based gene editing technology theoretically allows for precise manipulation of any genetic target within living cells, achieving the desired sequence modifications. This revolutionary advancement has fundamentally transformed the field of biomedicine, offering immense clinical potential for treating and correcting genetic disorders. In the treatment of most genetic diseases, precise genome editing that avoids the generation of mixed editing byproducts is considered the ideal approach. This article reviews the current progress of base editors and prime editors, elaborating on specific examples of their applications in the therapeutic field, and highlights opportunities for improvement. Furthermore, we discuss the specific performance of these technologies in terms of safety and efficacy in clinical applications, and analyze the latest advancements and potential directions that could influence the future development of genome editing technologies. Our goal is to outline the clinical relevance of this rapidly evolving scientific field and preview a roadmap for successful DNA base editing therapies for the treatment of hereditary or idiopathic diseases.

## Introduction

**Fig. 1:**
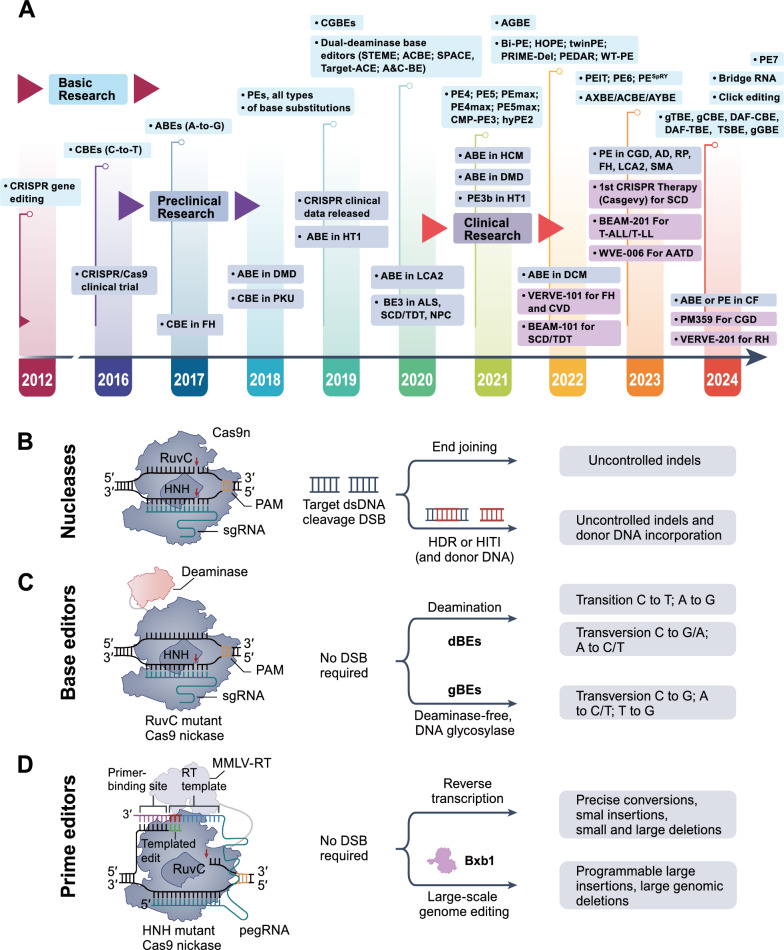
CRISPR-Cas9 technology for precision genome editing. **A** Timeline of key research reports on CRISPR-based base editing and prime Editing. **B** Cas nucleases can induce target DNA disruption by forming insertions or deletions (indels) or through DNA integration, which is often accompanied by a substantial amount of undesired indel byproducts. **C** Currently, two types of DNA base editors can achieve base transitions and transversions: deaminase-based base editors (dBE) and non-deaminase-dependent glycosylase-based base editors (gBE). The former category includes ABE, CBE, DdCBE, A&C-BEmax, AYBE, AXBE/ACBE, and CGBE, et al., all of which necessitate the deamination of A or C. The latter group comprises glycosylase-based guanine base editors (gGBE), thymine base editors (gTBE), and cytosine base editors (gCBE). **D** Prime editing can be programmed for any type of precise nucleotide substitution, and for insertions or deletions spanning up to several hundred bases. Prime editors typically employ Cas9 nickase, which incises only the non-complementary strand. The red DNA indicates the precisely edited sequence, while the yellow DNA bases show the location of the protospacer adjacent motif (PAM) essential for Cas9 targeting. RuvC and HNH are the nuclease domains of Cas9. HDR stands for homology-directed repair; HITI for homology-independent targeted integration. MMLV-RT, Moloney murine leukemia virus reverse transcriptase; pegRNA, prime editor guide RNA; PAM, protospacer adjacent motif

Programmable genome editing tools have revolutionized biomedicine, driving biotechnological innovation and demonstrating the potential for treating genetic diseases. Among these, RNA-programmable CRISPR systems have quickly become the forefront of this revolution due to their remarkable efficiency, ease of programmability, and wide range of applications [1–4]. These advancements have led to the development of three distinct technologies: CRISPR-associated nucleases, base editors, and prime editors. These tools enable genome editing across various targets and cell types in mammalian cells, each with differing capabilities and limitations that determine their optimal application in precise genomic manipulation. Over the past decade, through functional metagenomics, scientists have discovered more CRISPR enzymes and systems, significantly broadening the applications of CRISPR editing and providing the scientific community with a versatile and adaptable set of tools to explore biological functions, analyze gene interactions, and develop strategies for tackling human diseases and improving crops. With the approval of the first CRISPR-based human therapy (CASGEVY™), CRISPR genome editing has entered a new era from the laboratory to clinical application (Fig. 1A).

## CRISPR–Cas nuclease-based genome editing

The Cas nucleases, such as Cas9 or Cas12, can be redirected to different genomic sites by designing single guide RNAs (sgRNAs), thereby generating double-strand DNA breaks (DSBs) at specific target sequences [5, 6]. Since nucleases cannot directly modify DNA sequences, cells typically repair these breaks via end-joining pathways or through precise homology-directed repair (HDR) mechanisms [7, 8]. Although non-homologous end joining (NHEJ) can effectively rejoin the two ends of a DSB, it is error-prone and often results in insertions or deletions (indels), with many indels leading to frameshift mutations in coding sequences. Therefore, Cas nucleases are particularly suitable for gene disruption [8]. Moreover, these indels are often unpredictable and challenging to control precisely [9–12]. In contrast, HDR is a precise DSB repair pathway that relies on the presence of homologous DNA molecules to guide the repair, allowing for the accurate introduction of desired mutations, insertions, or deletions at the target genomic site [13, 14]. Although this method theoretically enables precise nucleotide editing, HDR is predominantly active in mitotic cells [15–17], making it ineffective in most therapeutically relevant cell types, and it is often supplanted by end-joining mechanisms during DSB processing, leading to a complex array of editing outcomes. In homology-independent targeted integration (HITI), non-homologous DNA donors are inserted at DSB sites via NHEJ. However, this method cannot control the direction and number of insertions, and the unintended indel outcomes often outweigh the expected precise corrections [18]. Additionally, off-target cuts generated by nucleases can lead to genomic rearrangements such as deletions, inversions, or translocations, triggering DNA damage and stress response pathways [19–26], thereby increasing the risk of oncogenesis (Table 1). Consequently, alternative CRISPR technologies are being developed to enhance precision and versatility, aiming for precise gene correction without generating DSBs (Fig. 1B).

**Table 1:** Features of gene editing tools

	Cas9 nuclease	Base editors	Prime editors
Components	Cas9; SgRNA; donor DNA	Cas9 (H840A) nickase-cytidine Deaminase/adenosine deaminase fusion protein; SgRNA	Cas9 (H840A) nickase-reverse Transcriptase fusion protein; pegRNA; nSgRNA
DNA break	DSB	SSB	SSB
Cell cycle dependence	YES	NO	NO
Principle	NHEJ/HDR/MMR	BER, MMR	Reverse transcription, MMR
Cellular toxicity	DNA DSB-associated cellular toxicity	Minimal cellular toxicity	Minimal cellular toxicity
Editing types	Indels, large deletions, chromosomal translocations	Single nucleotide transition or transversion	Small insertion, deletion or base conversion
Off-target effects	Stochastic indels	Indels; bystander edits	Relatively low indels
Pros	High efficiency; easy to multiplex; many proven options for viral and non-viral delivery ex vivo and in vivo; stochastic indels are predictable	Single base conversion with good eficincy; aplicable to genome and transcriptome; low indels	High product purity compared with Cas9+ HDR; no DNA template required; dual pegRNA approaches (HOPE, PRIME-Del, TwinPE, GRAND, Bi-PE, PEDAR) enable insertions up to 250 bp and deletions up to 10 kb
Cons	Off-target indels, Requires a DNA template; low efficiency; indel byproducts; cell-cycle dependent	Dependent on fixed distance to PAM; bystander edits, off-target deamination	Low efficiency; reverse transcriptase has no proofreading activity
Pipelines (examples)	Exa-cel (TDT/SCD), EDIT-101 (LCA10), EDIT301(TDT/SCD), NTLA-2001(ATTR), NTLA-2002(HAE), nula-cel (SCD), EBT-101(HIV-1)	VERVE-101 (LDL-C), VERVE-201(LDL-C/TRL), BEAM-101 (SCD/β-thalassemia), BEAM-102 (SCD)	PM359 (CGD)

## Precise genome editing

### Base editors and optimization

Base editing is one such versatile technology that enables targeted point mutations without the need to generate DSBs or DNA donor templates, thus facilitating editing in HDR-deficient cells. Base editors (BEs) are modular fusion proteins comprising a catalytically impaired Cas9 nickase (a version of Cas9 with an inactivated RuvC domain) [6, 27–29] fused to a nucleotide deaminase enzyme [30–35], enabling the conversion of one base to another (Fig. 1C). In base editing, guide RNA directs the base editor to a specific genomic DNA sequence, where the Cas protein displaces the target single-strand DNA, allowing the specific deaminase to catalyze deamination of the ssDNA. Two forms of base editors were initially developed: cytosine base editors (CBEs) and adenine base editors (ABEs) [30–34]. CBEs contain a catalytic domain from cytidine deaminase (such as APOBEC1) and an inhibitor of uracil glycosylase (UGI) domain, mediating the conversion of C to T [30]. ABEs use an adenine deaminase domain from tRNA-specific adenosine deaminase (TadA), which has been engineered through directed evolution to act on single-strand DNA (ssDNA), resulting in A-to-G conversion [32]. Compared to Cas nucleases, base editors exhibit higher efficiency and produce fewer indel byproducts, with significantly reduced DSB-associated adverse outcomes [19, 21, 23–26, 36, 37]. Since their invention, the initial CBE and ABE editors have undergone multiple design iterations to enhance activity and reduce off-target deaminase-induced editing [27, 38–45]. The types and scope of base editing have also expanded, with some base editors combining cytidine and adenine deaminase in one [46–48], and swap-editing types such as C-to-G or A-to-C and T-to-C or T-to-G [49–55]. It is noteworthy that the efficiency and purity of swap-type base editing vary depending on the target [49, 51, 52, 54]. Due to the predictability of editing outcomes, base editors (BEs) have been employed in whole-genome gene knockout and mutation screening studies [56]. Base editors, with the potential to correct single-point pathogenic genes or introduce protective mutations, have gradually been applied in clinical settings, including several studies conducted in non-human primates [57–59]. Despite the precise control base editors offer over editing outcomes, undesired base editing results are sometimes observed as more active base editors are developed. These effects can be classified based on whether they occur at on-target or off-target sites [60], and whether they are dependent on Cas9 [6, 61–66]. The widespread use of base editors has necessitated continuous performance optimization and improvement [6, 67, 68]. Major areas of exploration in base editor development include reducing the occurrence of undesirable editing byproducts [30, 38, 39, 43, 60, 69–75], enhancing editing activity and purity [67, 68, 76], expanding the range of genome-targetable bases [6, 27, 39, 41, 77–80] and diversity [80, 81], and improving editing precision [30, 32, 38, 70–75]. Although these engineering improvements have mitigated many deficiencies [27, 38–45], they have not yet achieved all 12 potential point mutation substitution types [55, 82, 83], and are unable to perform other types of DNA editing such as insertions, deletions, and most types of conversions (Table 1).

### Prime editors and optimization

Prime editing meets the need for a precise and highly flexible gene-editing technology capable of achieving a variety of DNA replacements, small-scale insertions, and deletions at specific target sites in the genome of living cells without directly forming double-strand DNA breaks (DSBs) [84] (Fig. 1D). Prime editors rely on a protein component and a prime editing guide RNA (pegRNA), with the former composed of a Cas9 nickase domain (with the HNH nuclease domain deactivated) and an engineered reverse transcriptase domain. The latter specifies the target site and contains a programmable RNA template for the desired DNA sequence change. To mediate editing, PE makes a nick at the target genomic site, releasing a segment of genomic DNA. Under suitable base pairing conditions, this segment can hybridize with the primer binding sequence. After hybridization, this genomic fragment acts as a primer for reverse transcriptase, which uses the extended sequence of pegRNA as a template for synthesis. The genome ends formed by reverse transcription can be reintegrated into the genome through end-repair processes [84]. Instead of relying on deaminases to chemically alter bases, PEs rewrite the target DNA using reverse transcriptase guided by the “prime editing guide RNA.” Since PEs modify the genome through three independent hybridization events, namely, prime editor binding and cutting at the target site complementary to the pegRNA spacer, hybridization of the pegRNA’s primer binding site (PBS) to the 3ʹ end of the cleaved target DNA, and hybridization between the synthesized reverse transcription template and the genome, this demand for multiple base pairing events greatly enhances the flexibility, editing purity, and DNA targeting specificity of prime editing compared to base editing and HDR. To date, prime editing has been successfully applied in a variety of organisms [85–89] and tissue types [90–92] (Table 1).

However, prime editing efficiency varies widely and is often low depending on the editing purpose, target site sequence, and cell type. To address these limitations, various methods need to be developed to improve PE efficiency and safety in different cell types. In fact, since the design of the first-generation prime editors [84], recent advances in understanding the cellular determinants of prime editing efficiency have led to several improvements: 1. Optimization of pegRNAs [93–96], such as adding stable secondary structures to the 3ʹ end of pegRNAs to resist degradation [97]. Additionally, current efforts are defining the rules for pegRNA design. With ongoing improvements in computational and AI-based tools, researchers can more easily plan and conduct gene editing experiments in different biological contexts, considering cell types and sequence contexts [98–107]. 2. Refining the prime editor protein [85, 96, 108–111], such as optimizing nuclear localization sequences [108, 112–114]. Furthermore, phage-assisted protein evolution and engineering can create shorter, more efficient constructs like PE6 [115]. Notably, smaller reverse transcriptase domains are particularly useful, as delivering large gene editors in these contexts poses a challenge [113, 116–118]. 3. Manipulation of DNA repair pathways, such as inhibiting mismatch repair pathways [94, 111, 119, 120]. Recently, genome-wide CRISPRi screening identified a key cellular determinant of prime editing—La, a small RNA-binding exonuclease protective factor. Fusing La with prime editing proteins to create PE7 significantly improved prime editing efficiency [121]. A deep understanding of the parameters influencing the formation of prime editor protein-pegRNA-genome site complexes in various cellular environments could provide crucial insights for future iterative improvements of prime editing technology [97, 119, 122].

Additionally, Prime editing strategies using multiple pegRNAs have expanded the potential size of sequence insertions [123, 124]or deletions [124–128]. Moreover, combining prime editors with site-specific recombinases has enabled the first RNA-programmed gene insertions or swaps of gene-sized fragments (> 5 kb) in mammalian cells [123, 124, 129] (Table 1).

Prime editors are complex molecular machines that generate diverse intermediates that must be resolved by cellular repair mechanisms to achieve successful editing. Cas effectors, reverse transcriptase, pegRNA components, and innovative gene editing strategies all contribute to enhancing the functionality of these systems. Understanding how these modifications affect the various stages of the prime editing process and related DNA repair processes is critical. Additionally, prime editing efficiency is strongly influenced by the cis chromatin landscape and is particularly promoted by active transcription. CRISPR-mediated transcription activation (CRISPRa) can be used to enhance prime editing efficiency, which may be useful in both basic research and disease treatment [130]. Computational and experimental approaches help narrow down the search for optimal components, including optimizing the relatively high error rate of reverse transcriptases lacking proofreading activity [131], which must be handled with caution in clinical applications. Future challenges include optimizing the delivery of large gene editors while minimizing genome damage. As prime editing technology continues to improve and develop toward clinical translation, the coming years promise to be exciting.

## Methods for delivering genome-editing tools

Effectively delivery and expression of editing components within target cells are crucial for achieving efficient genome editing, requiring a delicate balance between optimizing target editing efficiency and minimizing off-target effects. When selecting the optimal delivery vector for therapeutic genome editing, it is essential to comprehensively evaluate the impact of various biological and molecular barriers on intracellular macromolecule delivery, as well as how delivery strategies influence the specificity of genome editing (Fig. 3A). These considerations include: (1) selecting the type of cargo to be delivered (DNA, mRNA, or RNP) and tailoring the delivery route according to the therapeutic objective, such as gene knockout or inducing target mutations to reverse disease-associated single-nucleotide variants (SNVs); (2) employing encapsulation vectors capable of achieving targeted binding to desired cells in different target environments, such as the central nervous system or liver [132]; (3) ensuring that the cargo is released into the appropriate intracellular compartment after crossing the cell membrane; (4) avoiding potential therapeutic immune responses; and (5) effectively controlling and regulating base editor expression [81, 133]. Over the past decades, researchers have developed and engineered a variety of delivery vectors that meet these criteria and have successfully applied them across multiple organisms [134–136].

### Direct nucleic acid transfection and viral delivery

Viruses offer several advantages for delivery, including efficient transduction and tissue specificity, making them attractive modalities for in vivo delivery of gene editors (Table 2). Lentiviral transduction of individual pegRNAs has been demonstrated to enable the screening of pooled pegRNA libraries [108, 123, 128, 137]. Lentiviruses can randomly integrate DNA encoding base editors (BE), prime editors (PE), and seRNA/pegRNA into the genome, achieving high editing efficiency in human cell lines, induced pluripotent stem cells (iPSCs), and mouse cortical neurons [84, 126–128, 138–140]. However, due to insertional mutagenesis, lentiviruses may increase the risk of tumorigenesis and off-target editing from prolonged expression [141]. To mitigate these risks, transient expression is preferred, making it more favorable for therapeutic applications. Among DNA viruses with lower integration risks, adeno-associated viruses (AAVs), adenoviruses, and herpes simplex viruses (HSVs) have been successfully employed for transient transduction [141].

**Table 2:** Overview of methods for delivering genome-editing tools

Delivery vehicle	Categories	Cargo types	Technology introduction	Benefits	Challenges	Refs.
Viral	Adeno-associated virus (AAV)	DNA	Single-stranded Capsid diameter (~ 25 nm); DNA without an envelope; Maximum payload size is 4.7 kb; Viral entry via cell surface receptors	High transduction rate; Non-pathogenic virus; A variety of engineered serotypes are available	Immunogenic, Limited packaging capacity Pre-existing immunity to natural serotypes; Prolonged expression Leads to risk of off-target editing	[268, 292]
	Lentivirus (LV)	DNA RNA	Single-stranded Capsid diameter (~ 100 nm) Viral entry via cell surface receptors	Large cargo size, broad targeting and pseudo-typing	Immunogenic, Limited efficiency in vivo; Non-specific DNA integration creates an oncogenic risk	[137, 266, 368]
	Adenovirus (AdV)	DNA	Non-enveloped Capsid diameter (~ 100 nm) Double-stranded DNA virus, ~ 8–36 kb maximum packaging limit; Viral entry via cell surface receptors	Large cargo size	Immunogenic, Prolonged expression leads to risk of off-target editing	[229, 254]
	Retrovirus	DNA	Single-stranded; Capsid diameter (100–200 nm) ~ 3 kb maximum packaging limit; Broad class that includes lentivirus; Viral entry via cell surface receptors	Large cargo size, broad targeting and pseudo-typing	Non-specific DNA integration creates an oncogenic risk; immunogenic	[368, 369]
Non-Viral delivery strategies	Electroporation	DNA, mRNA Protein	Direct delivery to the cytoplasm via membrane disruption	Very efficient, widely applicable to all types of cells; Minimal size restriction	Specialized equipment required Low throughput, only practical for ex vivo therapeutics	[230, 301, 315]
	Direct administration	DNA, mRNA	Systemic delivery of naked DNA or RNA	Simple procedures and administration	Immunogenic, only works for targeting hepatocytes, Mostly ends up in the liver	[124]
	Cell injection	DNA, mRNA	Injection directly into the cell nucleus or cytoplasm	Effective	Extremely low throughput and viability	[206, 208, 286, 370]
	Cationic lipid	mRNA, DNA, RNP	Cargoes are released by endocytosis and endosomal escape after being encapsulated in cationic lipids or lipid spheres	Many formulations; Relatively easy production; Off-targets are reduced by controlling the dose and time of the base editor	Immunogenic Mostly ends up in the liver	[188, 371]
	Lipid nanoparticles (LNP)	mRNA, DNA, RNP Protein	Cargoes are released by endocytosis and endosomal escape after being encapsulated in lipid nanoparticles or lipid spheres	Many formulations; Relatively easy production; Off-targets are reduced by controlling the dose and time of the base editor	Immunogenic Mostly accumulate in the liver	[58, 255, 298]
	Virus-like particles (VLP)	mRNA, DNA, RNP	Protein cages derived from viruses or created computationally	Minimized off-target editing; Engineerable protein-based scaffold	Immunogenic Low in vivo efficacy; No extensive experience in clinical trials	[181, 372]
Others (Emerging or potential strategies for genome-editing tools delivery)	DNA cages	RNP	Particles built using DNA origami	Engineerable DNA-based scaffold	Underexplored Immunogenicity remains to be tested in vivo	[187, 373, 374]
	Cell-penetrating peptides (CPP)	DNA mRNA, RNP	Peptide fusions that allow cells to enter cargo	Simple implementation	Preliminary technology not applied to genome-editing	[182, 184]
	Physical	DNA, mRNA, RNP	Physical disruption of cells employing nanowires, hydrostatic injection, or cell squeezing to assist base editors in entering the cell	Physical approaches circumvent endosomal entrance routes	Cell damage, poor delivery controllability, cell type restriction; immunogenicity	[137, 234]
	Extracellular vesicle (EVs)	DNA, RNP	Cargoes release occurs through outward budding of the plasma membrane (micro-vesicle pathway) or inward budding through endosomal membranes (exosome pathway)	High biosecurity	Nonspecific packaging of contaminant proteins/RNA; High variability in composition between producer cells	[375]
	Engineered Contractile Injection System (eCIS)	mRNA, DNA, RNP Protein	Redesigning the tail fibers of injection-like machines to deliver target proteins/drugs to target cells	Precision therapy, expected to target all types of cells Fewer side effects	Preliminary technology, not applied to genome-editing	[185, 376]

Due to their low immunogenicity and broad tissue tropism, AAVs are particularly promising tools for delivering gene editors. They can transport their payloads to various clinically relevant tissues, including the eyes, liver, brain, heart, and muscles [142–146]. Different naturally occurring AAV capsid serotypes can target different tissues in vivo, further enhancing AAV's versatility [146]. Recent studies have shown that coupling selected AAV capsid proteins with the human transferrin receptor (TfR1) allows crossing the blood–brain barrier, enabling effective gene delivery within the central nervous system [147]. AAV has been utilized in FDA-approved drugs [148, 149] and therapies in clinical trials [150]. However, AAV can only package approximately 4.7 kilobase pairs (kb) of DNA cargo, which is smaller than the size required for PE editors and pegRNA (7 kb).

To overcome this limitation, multiple research teams have split genome editors into two protein halves, each fused with split inteins or trans-splicing elements [113, 114, 116, 117, 151]. Upon co-infection with AAVs expressing each PE-intein half, the full-length genome editor is reconstituted through trans-splicing. In another approach to bypass the AAV packaging limit, Kim and colleagues encoded each genome editor half on transfer RNA splicing AAVs (tsAAVs) [137]. Homologous recombination between inverted terminal repeat (ITR) sequences of tsAAVs generates full-length transcripts, leading to PE expression. Finally, Sontheimer and colleagues demonstrated that delivering unlinked nCas9 and RT (sPE) via two different AAVs can achieve high-efficiency genome editing [96]. Encouragingly, these strategies have induced moderate editing in mouse liver and retina. Similar split-CBE/split-ABE approaches have been used to treat diseases such as ALS, phenylketonuria, and Niemann–Pick type C, as well as to correct hereditary hearing loss in mice by editing genes in the brain, liver, retina, heart, skeletal muscle, and inner ear [152–157]. Although current dual-AAV PE delivery shows lower editing efficiency compared to dual-AAV Cas9 nuclease or BE delivery, ongoing advancements in optimizing PE proteins and pegRNAs are expected to significantly enhance in vivo prime editing efficiency [97, 119].

Another approach for delivering the editing machinery involves using a single AAV vector, which can be facilitated by smaller Cas homologs. For example, the smaller gene size of SaCas9 allows it to be packaged within a single AAV alongside one or two sgRNA expression cassettes, making it a common choice for single-AAV strategies. The discovery of compact Cas9 variants, such as SauriCas9 [158], CjCas9 [159], and Nme2Cas9 [160], as well as the use of smaller nucleases like Cas12f [161] and Cas12j [162], has also been reported. Notably, these Cas proteins contain only 400–700 amino acids, further expanding the number of Cas9 enzymes that can be packaged into a single AAV vector. This approach is preferable as it reduces the required AAV dosage and offers improved safety compared to dual-AAV systems [163]. Importantly, these systems are small enough that adding a base editor domain still allows for a single-AAV format, and single vectors delivering in vivo base editing have subsequently been reported [163, 164].

Additionally, controlling the expression of gene editing agents delivered by AAV can maximize gene editing specificity and enhance the safety of future therapeutic applications [165–168]. Despite AAV's relatively low immunogenicity, the host can still generate antibodies against the AAV capsid and its delivered gene, posing challenges for therapies requiring multiple doses of base editors. Minimizing the long-term expression of these agents offers a useful strategy for improving safety and enhancing gene editing efficacy [169–172].

In comparison to AAV, adenoviruses and lentiviruses present distinct differences and challenges (Table 2). Adenoviruses and lentiviruses induce a more pronounced inflammatory response in vivo, raising concerns about their safety in clinical applications [141]. However, both viruses have unique advantages, such as their larger packaging capacities—up to 40 kb and 10 kb, respectively. This feature allows them to encapsulate more gene-editing elements, such as prime editors and pegRNAs, in a single vector, thereby improving transduction efficiency. Moreover, adenoviruses efficiently transduce a variety of tissues, while lentiviruses facilitate semi-random integration into the host cell genome. These characteristics have been leveraged in studies such as those by Schwank and his team, who achieved up to 60% gene editing efficiency in mouse liver using adenovirus vectors, surpassing the dual-AAV system [113]. Additionally, researchers like Gonçalves have achieved efficient gene editing in cell cultures using high-capacity adenovirus vectors [173]. Despite the promising outcomes of these strategies, optimizing viral vectors for in vivo PE transduction is critical for achieving therapeutic genome editing in human patients [141]. While these studies highlight the potential of adenoviruses and lentiviruses in gene editing, their sensitivity to innate immune responses and the risk of random integration still limit their efficiency and efficacy in vivo. Combining viral and non-viral delivery methods may offer a promising solution by leveraging the strengths of both approaches to optimize efficiency and safety.

### Non-viral delivery

A common non-viral delivery strategy involves introducing DNA encoding genome editing components into target cells, relying on the cell's endogenous transcription and translation mechanisms to produce BE/PE RNPs. In various mammalian cell lines, transient lipid-mediated transfection or electroporation of BE/PE plasmids can achieve high editing efficiency, and selecting appropriate cell types can further optimize editing outcomes [84, 119, 174]. Additionally, hydrodynamic tail vein injection of plasmid DNA has been shown to effectively deliver BE/PE to hepatocytes in mice [96, 114, 116, 125, 137]. These non-viral nucleic acid delivery techniques induce transient expression of genome editors in mammalian cells, minimizing unintended off-target edits (Table 2).

mRNA delivery allows for rapid protein expression without the need for transcription, followed by relatively quick degradation, thereby eliminating the possibility of DNA integration into the genome [175]. By shortening the duration of genome editor expression, mRNA delivery reduces the chance of off-target editing. However, chemical modifications are necessary to maintain the required activity levels and prevent immune responses induced by mRNA [176]. Studies have shown that introducing PE mRNA along with chemically modified or in vitro transcribed guide RNAs via electroporation can achieve efficient prime editing in cultured cell lines, primary human T cells, and human pluripotent stem cells, often outperforming DNA transfection [96, 119, 174, 176–178]. Additionally, the PE4Max and PE5Max editing systems are particularly well-suited for mRNA delivery, limiting the mutagenic risk associated with the MLH1dn component in their composition [119]. Therefore, RNA delivery may be an effective strategy for prime editing in cell cultures, embryos, and hematopoietic cells ex vivo.

Delivering genome editors as pre-assembled RNPs is the most effective method, as it enables the fastest cellular response while reducing cytotoxicity. RNP delivery may be more efficient than mRNA-based delivery, offering a faster onset of editing. Specifically, delivering PE-pegRNA ribonucleoproteins (RNPs) reduces the time target cells are exposed to PE reagents, minimizing off-target editing. Additionally, RNP delivery of PEs bypasses the need for intracellular transcription and translation mechanisms to produce PEs. For instance, Yeh and colleagues purified the PE2 protein complexed with pegRNA and microinjected these RNPs into zebrafish embryos, achieving moderate editing effects [179]. Despite the theoretical advantages of RNP delivery, current methods for PE RNP delivery still lag behind DNA and mRNA delivery in terms of efficiency [96, 119, 177] (Table 2).

### Novel delivery methods

Recent developments in novel lipid nanoparticle (LNP) formulations have enabled tissue-specific targeting, providing a versatile approach for packaging and delivering CRISPR components, while enhancing cellular uptake and reducing off-target editing [180] (Table 2). Additionally, the emergence of fourth-generation engineered viral-like particles (VLPs) has been driven by improvements in cargo release, precise loading, and stoichiometry. These VLPs can deliver genetic payloads to the liver following systemic administration and target the eyes or brain after localized delivery [181]. Cell-penetrating peptides have also shown great potential as delivery tools for CRISPR enzymes, particularly in editing primary human lymphocytes [182], neural cells [183], and airway epithelial cells [184]. Furthermore, redesigned bacterial contractile injection systems have enabled the efficient and specific transient delivery of protein cargos, such as genome-editing nucleases [185, 186]. Engineered viral-like particles, which mimic viruses but lack viral genetic material, have also emerged as powerful alternatives for in vivo applications [181]. With advancements in computational protein design, newly engineered protein cages are being developed to modularly deliver gene-editing complexes to biological environments that are typically challenging to access [187].

Despite the promise these technological advances hold, it is crucial to rigorously evaluate their cytotoxicity, off-target effects, and immunogenicity across various cell types and tissues. Moreover, the scalability of these methods for clinical applications, along with the high costs associated with their development and manufacturing, presents significant challenges. Further details on delivery strategies for editing formulations have been extensively discussed in previous reviews [188, 189].

## DNA base editors in basic research and medicine

**Fig. 2:**
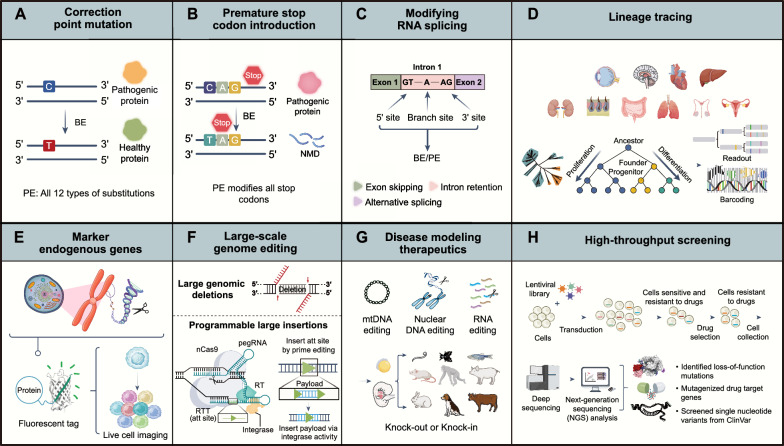
Applications of DNA base editors related to human health. **A** The correction of single-point mutations encoding pathogenic proteins. **B** The introduction of premature stop codons to eliminate pathogenic transcripts through the NMD surveillance pathway. Prime editing is capable of performing any potential stop codon corrections and formations. **C** The splice donor, acceptor, and branch point can be edited with BE and PE tools to modify splicing. **D** Base editing barcodes create new possibilities for efficient single-cell lineage tracing by recording cell division events and long-term marking of genetic mutations. **E** BEs and PEs can be used for base substitutions at specific sites in response to external or internal stimuli, recording molecular events triggered by these stimuli. Additionally, PEs can insert fluorescent tags or cellular localization signals, making them highly versatile for selective detection or manipulation of proteins within cells. **F** Prime editing with paired prime editor guide RNAs (pegRNAs) can efficiently mediate small edits or large insertions and deletions. Prime editing with serine integrases enables the targeted insertion of gene-sized (> 1 kb) DNA fragments. First, prime editing installs an attB site in the genome, followed by the integration of donor DNA into this site with Bxb1 recombinase. **G** Base editors and prime editors have advanced the generation of specific genetic alterations, including gene knockouts, gene insertions, and point mutations in cultured cell lines and animals, thereby allowing the modeling of human genetic disease variants. **H** These applications involve high-throughput gene function analysis through guide RNA libraries. Cells transduced with these libraries undergo selection-based assays, and the enrichment or depletion of guide RNAs in the cell population is analyzed with next-generation sequencing technologies, thereby identifying genes linked to specific biological processes

Advances in genome editing technologies have led to a wide range of applications, encompassing breakthroughs in basic research to the development of new therapies, with immense potential to improve human health. Both base editors (BEs) and prime editors (PEs) can not only correct mutated genes (Fig. 2A) but also be widely applied across various functions.

### Gene expression regulation

Cytosine base editors (CBEs) can inhibit gene expression by introducing premature termination codons (PTCs) [190, 191] (Fig. 2B). The i-Silence strategy of ABEmax silences target genes by converting ATG into GTG or ACG, effectively avoiding the production of truncated protein variants [192] (Fig. 2B). Additionally, base editors can precisely regulate the expression of specific isoforms. Most proteins exist in multiple isoforms, and alternative splicing of pre-mRNA is a key step in determining their type. The CRISPR-SKIP method takes advantage of the fact that most introns end with a G [193]. By editing Cs on the complementary strand, CRISPR-SKIP converts the G within the splice acceptor site to an A, thereby affecting the splicing process and altering the composition of the mature transcript. Due to their high accuracy and simplicity in single-nucleotide editing, DNA base editors have also been used for functional screening of single-nucleotide variants. Notably, about 90% of human pathogenic gene variants are single-base mutations or involve insertions and deletions of fewer than a dozen base pairs [194]. All of these types of DNA changes fall well within the capabilities of the prime editing systems (Fig. 2C).

### Molecular recording and genealogical tracking

Genome-wide gene knockout screens and other genetic approaches are powerful tools for studying biological functions in health and disease. Leveraging the advancements in genome editing technologies, CRISPR screening can systematically disrupt thousands of individual genes and non-coding genomic elements within cells, aiding in the identification of genes associated with specific biological pathways and their interactions. Typically, cellular events or lineages are combined with gene editing at target sites, which serve as records that can be decoded through sequencing. Compared to barcodes derived from NHEJ, the barcodes generated by base editing can record more mutation information, as base editing slows down target site depletion, which is particularly beneficial for long-term lineage-tracing experiments [195–200] (Fig. 2D). Unlike Cas9 nucleases and base editors, prime editing can directionally insert DNA information that encodes the type, duration, and sequence of cellular signals [201]. To achieve directed transcriptional activity, PE-mediated short insertions not only encode the cell signals in the record but also complete the protospacer sequence of the adjacent target site, making the adjacent site a valid base editing target for the next recording event. These studies demonstrate the tremendous potential of molecular recorders based on base editing in biotechnology and basic research.

### Marker endogenous genes

The ability to tag endogenous genes is widely utilized for the selective detection or manipulation of intracellular proteins. For instance, the prime editing system can be programmed to insert short tags like FLAG [84, 97], as well as similarly sized tags such as HiBiT luciferase [202], GFP1 [203], or cellular localization signals. Additionally, next-generation prime editing systems have been demonstrated to install larger tags (Fig. 2E); for example, PASTE has successfully fused GFP into the reading frame of genes such as ACTB [123]. These findings showcase the capability of prime editing to install protein tags or peptides on endogenous genes. Enhancing the efficiency of PE for long-fragment insertions will further expand the applications of gene tagging (Fig. 2F).

### Animal model

The genetic modification of model organisms plays a crucial role in uncovering the functions of genes and the biomolecules they encode within complex systems. One of the primary advantages of base editors lies in their ability to introduce pathogenic point mutations with precision and efficiency, which account for the majority of known human genetic variations [194]. This capability is particularly valuable in generating cell-based homologous disease models. By editing these cells and directing their differentiation into specific cell types affected by the disease, researchers can study diseases within the relevant cellular context. To date, base editors have significantly contributed to modeling human genetic diseases across various systems [134, 204–211], accelerating research progress from basic science to drug discovery and targeted therapeutic interventions (Fig. 2C). Compared to HDR using CRISPR-Cas nucleases, prime editing offers higher editing purity and greater flexibility than base editing, making it highly suitable for creating transgenic organisms with precise genomic alterations [179, 212–214].

### Functional screening of genetic variants

Introducing genetic perturbations and assessing their impact based on functional characteristics or enrichment is a common method for elucidating biological pathways and mechanisms. Cas nucleases have been widely used for gene knockouts in pooled and arrayed screens [215, 216]. However, the low efficiency and product purity when using Cas nucleases in combination with HDR in many cell types limit their application in screening. Base editors, due to their ability to introduce random base substitutions at target sites, have proven practical for saturating mutations in genes of interest [56, 217, 218]. Additionally, base editors have shown great potential in large-scale functional analysis of nucleotide variants and drug discovery [70, 218–222] (Fig. 2H), but their application is primarily limited to single-base conversion editing. Because of its precision and versatility, prime editing is particularly suitable for generating gene mutations in functional screens. In a proof-of-concept study, Cohn and colleagues used prime editing to assess genetic variants of unknown function in *NPC1*, the gene responsible for Niemann–Pick disease type C1, a lysosomal storage disorder [223]. Similar to the Random-PE strategy developed by Yao and colleagues [224], they used pegRNAs to introduce random codons (NNN), thereby inducing site-specific mutations. However, these methods identify DNA changes induced by prime editing by sequencing the edited loci, which often limits the mutation region to a single gene. In contrast, sequencing pegRNAs can recover the programmed prime editing characteristics without restricting the editing location. As prime editing systems continue to improve, they will further enhance genetic screens for gene variants or combinations of gene variants within multi-gene pathways.

## In vivo and ex vivo therapeutic applications

**Fig. 3:**
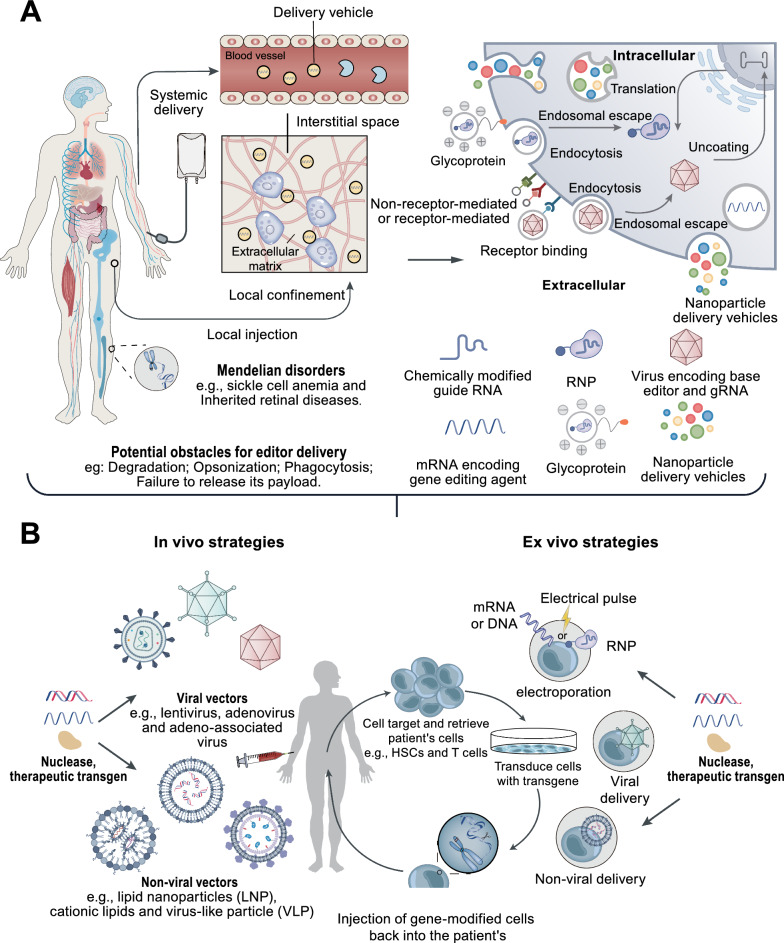
Overview of therapeutic genome editing delivery strategies. **A** Genome editing holds immense potential for treating monogenic and complex diseases with specific gene targets. Although in vivo delivery constructs are commonly used, ex vivo cell modification is also feasible. However, the successful delivery of biomolecules—including DNA, mRNA, single-guide RNA (sgRNA), proteins, or nanoparticles encapsulating these biomolecules—is hindered by extracellular barriers. These barriers include phagocytosis by macrophages or other phagocytic cells, enzymatic or hydrolytic degradation, and the induction of immune responses and cytokine production. Overcoming these extracellular barriers is crucial for the successful delivery of biomolecules and nanoparticles, which must subsequently be endocytosed by target cells. Once inside the target cells, the biomolecules and nanoparticles must escape from endosomes and localize to the cytoplasm (in the case of mRNA) or the nucleus (for DNA, sgRNA, or nuclease proteins) to enable effective gene editing. **B** The three primary delivery methods used for clinical genome editing are DNA, mRNA, and purified ribonucleoproteins (RNPs). These methods exhibit significant differences in key aspects, determining their suitability for editing specific cell or tissue types

The promise of therapeutic gene editing has inspired strong efforts to bring gene editing technologies to the clinic. Base editors and lead editors are ideal tools for correcting inherited diseases because of their high precision and product purity, and are widely used to treat many diseases with genetic components in various animal models. Some of these therapeutic gene editing strategies have entered clinical trials with promising early results [225, 226]. Currently, most current gene editing clinical trials involve ex vivo editing [225]. This is feasible for some key cell types (e.g., hematopoietic stem cells) [227], but most cell types are not suitable for ex vivo manipulation and transplantation into patients (Fig. 3B). In contrast, in vivo genome editing offers greater promise for the treatment of inherited diseases, with the promise of restoring endogenous gene regulation through a one-time treatment, thereby minimizing patient burden.

### Blood

**Fig. 4:**
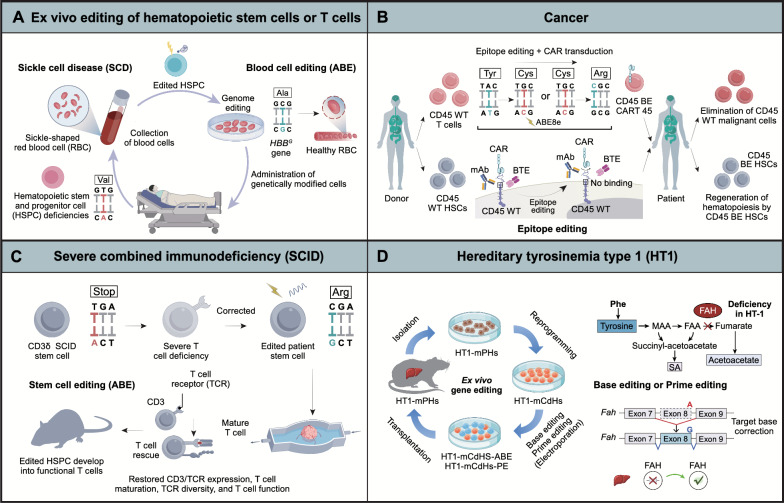
In vitro applications of precision genome editing technology for treating human diseases. **A** Blood disorders such as sickle cell disease may be treated by editing haematopoietic stem or progenitor cells (HSPCs) ex vivo, which then produce normal red blood cells (RBCs). **B** Epitope-edited haematopoietic stem cells and CAR T-cells fulfil the claim that anti-CD45 CAR T-cell therapy is both safe and effective, and this strategy is universally applicable to the treatment of haematological malignancies. **C** ABE can restore CD3δ expression and signaling in SCID patient HSPCs, allowing them to differentiate into functional T cells with diverse TCRs. When transplanted into a mouse model, these edited HSPCs can reconstitute the hematopoietic system and maintain corrected cells long-term. **D** Adenine base editing and prime editing of chemically derived hepatic progenitors rescue genetic liver disease

Ex vivo gene editing of patient-derived cells, followed by expansion and reinfusion, is an effective strategy for treating genetic hematologic disorders such as sickle cell disease (SCD) and transfusion-dependent β-thalassemia (TDT). These diseases are caused by mutations in the hemoglobin β subunit (HBB), leading to inadequate red blood cell function. Gene correction or reactivation of fetal γ-globin (HBG) expression can effectively treat these diseases, with the latter being more feasible. Research has shown that using a base editor (CBE) to edit the fetal hemoglobin promoter can induce compensatory fetal hemoglobin (HbF) expression in mice, thereby treating SCD and TDT [228, 229]. Moreover, the more active adenine base editor ABE8s, through editing the γ-globin promoter, achieves 60% efficient editing in CD34+ cells while avoiding off-target effects [81] (Fig. 4A).

Combining ex vivo gene editing with induced pluripotent stem cell (iPSC) technology may become a powerful genomic therapeutic strategy. For example, using the high-activity ABE8e to correct the SCD mutation in the β-globin gene in patient-derived CD34+ cells achieved 68% editing efficiency with minimal off-target mutations [230] (Fig. 4A). While hematopoietic stem cell transplantation (HSCT) can replace diseased cells with genetically engineered stem cells, current transplantation methods still have issues such as side effects and limited applicability [231]. To overcome these challenges, researchers designed lipid nanoparticle (LNP)-targeted mRNA encoding the ABE system to the hematopoietic stem cell (HSC) CD117 receptor, enabling in vivo editing via intravenous injection. This approach almost completely corrected the formation of sickle red blood cells in the hematopoietic system [232] (Table 3).

**Table 3:** Key examples of preclinical gene and cell therapies for base editing and prime editing

Year	Disease	Target gene	Editor	Delivery	Strategy	Therapeutic outcomes	Refs.
March 2020	SCD/TDT	*BCL11A*	BE3	Ex vivo via electroporation of RNP	Base editor-mediated modification of transcription factor binding sites	Engraftment of edited HSCs in NBSGW mice resulted in HbF induction	[377]
July 2020	SCD/TDT	*HBG1/2*	ABE8s	Electroporation in CD34+ human cells, ex vivo	Base editors targeted the *HBG1/2* promoters in HSCs	The target HBG1/2 promoter site in primary human CD34+ cells was edited with 60% efficiency	[81]
February 2021	SCD/TDT	*BCL11A*	ABE7.10max	Ex vivo delivery via adenovirus to hematopoietic stem cells in blood	Reactivation of fetal γ-globin in β-YAC mice	30% editing following selection, 21% of blood β-like globins were fetal hemoglobin	[229]
June 2021	SCD/TDT	*HBB*	ABE8e	Electroporation in CD34+ human cells, ex vivo	Conversion of the pathogenic sickle cell allele (*HBBS*) to a benign variant (*HBBG*)	Sixteen weeks post-transplantation, 68% of the β-globin in mouse blood was the edited form	[230]
July 2023	SCD/TDT	*HBB*	ABE	LNPs anti-CD117, in vivo	Directly modifying HSCs in vivo using mRNA-based delivery systems	Conversion of pathogenic E6V sickle cell variants to non-pathogenic variants with 88% editing efficiency	[232]
May 2022	SCD/TDT	*HBB*	PE3	Microinjection in mouse IVS-11–654 zygotes, ex vivo	Using PE3 to correct the IVS-II-654 mutation (C>T)	PE3 installed the correction (T>C), with an editing efficiency of 14.29%	[234]
April 2023	SCD/TDT	*HBB*	PE5max	HDAd5/35++ in mouse CD46/Townes, in vivo	Correct the *HBB* (c.20T>A; p.E6V) mutation	About 40% of βS alleles in HSCs were corrected, replacing 43% of sickle hemoglobin with adult hemoglobin on average	[235]
January 2021	HGPS	*LMNA*	ABE7.10max-VRQR	Split-intein dual-AAV9 in mouse *LMNA*, in vivo	Correct the *LMNA* (c.1824 C>T; p.G608G) mutation	30% editing in heart, 20% in aorta.2.4-fold increase in lifespan	[236]
November 2022	DCM	*RBM20*	VRQR-SpCas 9-ABEmax	Nucleofection in iPSCs, ex vivo	Correct the *RBM20* missense mutation (c.1906C>A; p.R636S)	92% efficiency of A-to-G editing	[245]
	DCM	*RBM20*	PE3b	Nucleofection in iPSCs, ex vivo		40% efficiency of A-to-C editing	
	DCM	*RBM20*	VRQR-SpCas 9-ABEmax	AAV9 in mouse *Rbm20R636Q*, in vivo		Improved heart function and longevity	
February 2023	HCM	*Myh6*	ABE8e	Split-intein dual-AAV9 in mouse 129SvEv, in vivo	Correct the heterozygous myosin R403Q variant associated with HCM	Achieved ≥ 70% ventricular cardiomyocyte expression and maintained durable, normal cardiac structure and function	[241]
February 2023	HCM	*Myh6*	VRQR-SpCas 9-ABEmax	Split-intein dual-AAV9 in mouse *Mylh6h403/h403*, in vivo	Correct the *MYH7* missense mutation (c.1208G>A; p.R403Q)	35% base editing rescues pathological manifestations of HCM	[239]
July 2017	HF	*Pcsk9*	BE3	In vivo via an adenoviral vector	Disrupting expression of *PCSK9* gene in liver	28% editing reduced PCSK9 protein expression	[144]
October 2018	HF	*Pcsk9*	BE3	In utero via an adenoviral vector	Disrupting expression of *PCSK9* gene in liver	Reduced plasma PCSK9 protein levels and reduced plasma cholesterol levels in mouse fetuses	[5]
January 2019	HF	*Pcsk9*	BE3	Adenovirus in mice, in vivo	Disrupting expression of *PCSK9* gene in liver	~ 20% editing reduced PCSK9 protein expression	[254]
August 2020	HF	*Pcsk9*	ABE	In vivo via functionalized lipid-like nanoparticles	Disrupting expression of *PCSK9* gene in liver	Reduced postnatal serum PCSK9 protein levels in mice	[255]
May 2021	HF	*Pcsk9*	ABE8.8	LNP in monkey, in vivo	Disrupting expression of *PCSK9* gene in liver	70% editing in mice and 67% editing in macaques reduced PCSK9 protein expression	[58]
May 2021	HF	*Pcsk9*	tBE-V5-mA3	AAV8 in mouse, in vivo	Created a premature stop codon	30% editing in mice reduced PCSK9 protein expression	[165]
May 2021	HF	*Pcsk9*	ABEmax	AAV8 and LNP encapsulation mRNA, in vivo	Disrupting expression of *PCSK9* gene in liver	67% editing in mice and 28% editing in macaques reduced PCSK9 protein expression	[57]
May 2023	HF	*Pcsk9*	v3em PE3	Split-intein dual-AAV9 in mouse, in vivo	Disrupting expression of *PCSK9* gene in liver	Achieved 39% editing 8 weeks after injection in mice	[118]
October 2018	PKU	*Pah*	SaKKH-BE3	In vivo via dual AAV vectors	Base-editor-mediated correction of nonsense mutation	Editing increased from 10% after 4 weeks to 25% after 26 weeks, with a return of blood phenylalanine to normal levels	[152]
January 2021	PKU	*Pah*	SaKKH-BE3	AAV8 and LNP encapsulation mRNA	Correct the disease-causing T-to-C mutation in exon 7	23% editing after 8 weeks using AAV.19% editing 1 week after second LNP dose. Return of blood phenylalanine to normal levels	[378]
March 2022	PKU	*Pah*	PE2^△RnH^	AdV in mouse *Pahenu2*, in vivo	Correct the *PAH* (c.835T>C; p.F263S) mutation	Correction efficiency of 11.1% (up to 17.4%), effectively reducing blood phenylalanine levels	[113]
			PE3^ΔRnH^	AAV8 in vivo		< 2% gene correction	
				Adenovirus in vivo		11.1% gene correction with < 0.2% indels	
February 2019	HTI	*Fah*	ABE6.3	Hydrodynamic injection in *Fahmut/mut* mice, in vivo	Base-editor-mediated splice site correction	Partially restored splicing, rapidly expanded Fah + hepatocytes in the liver, and rescued weight loss in adult *Fahmut/mut* mice	[257]
April 2020	HTI	*Fah*	Optimized ABE6.3	In vivo via lipid nanoparticle delivery	Base-editor-mediated generation of de novo in frame start codon	Rescued weight loss and restored Fah expression in liver tissue of *Fah* mutant mice	[379]
May 2020	HTI	*Fah*	BE4max	In vivo via dual AAV vectors	Base-editor-mediated generation of de novo in frame start codon	Restored Fah expression and halted weight loss in an *HTI* mouse model	[380]
August 2021	HTI	*Fah*	PE3	Hydrodynamic injection in *Fahmut/mut* mice, in vivo	Correct the *FAH* (c.706G>A) mutation	Edited hepatocytes in adult *Fahmut/mut* mice	[137]
May 2021	HTI	*Fah*	ABE max and ABE8e	Electroporation in HT1 CdHs mouse cells, ex vivo	Correct the *FAH* (c.706G > A) mutation in HT1-mCdHs	2.4% and 9.2% base editing in HT1-mCdHs	[261]
		*Fah*	PE3b	Electroporation in mouse HT1 CdHs, ex vivo		2.3% prime editing in HT1-mCdHs without any bystander effects	
October 2021	HTI	*Fah*	PEDAR	Hydrodynamic injection in *Fah*△*Exon5*mice, in vivo	Delete a ~ 1.38-kb pathogenic insertion in exon 5 of the *FAH* gene	With 8% correction rate	[125]
April 2022	HTI	*Fah*	split PE (sPE)	Dual-AAV8 delivery in *Fahmut/mut* mice, in vivo	Correct the *FAH* (c.706G>A) mutation	1.3% prime editing in *Fah*-mutant mice	[96]
February 2022	AATD	*SERPINA1*	CBE	LNP delivery in *PiZ* mice, in vivo	Correction of *SERPINA1* (p. Glu342Lys) mutation	Achieved up to 29% efficiency in correcting the pathogenic *PiZ* mutation	[381]
			ABE		Install a compensatory mutation (p. Met374Ile)	Achieved up to 31% efficiency in editing the target cytosine to introduce a compensatory mutation	
April 2021	AATD	*SERPINA1*	PE2	Hydrodynamic-mediated non-viral vector delivery.in vivo	Correct the *SERPINA1* (c.1024G>A; p.E342K) mutation	6.7% correction by hydrodynamic injection, 3% correction by dual AAV 10 weeks after treatment	[114]
			PE3	Dual AAV8 vectors		3.1% gene correction with 0.4% indels at day 70	
October 2020	LCA2	*Rpe65*	ABE7.10 max	Lentivirus delivery in rd12 mice, in vivo	Correct the *Rpe65* gene mutation on exon 3 (c.130C>T; p.R44X)	29% base editing, restored visual function	[266]
August 2021	LCA2	*Rpe65*	SpCas9-NG-PE2	Trans-splicing AAV2in rd12 mice, in vivo	Correct the *Rpe65* (p.R44X) mutation	The edited mice had better dark-adapted light-induced electrical responses	[137]
January 2021	LCA2	*Rpe65*	ABE7.10 max	AAV9 delivery in rd12 mice, in vivo	Correct the *Rpe65* gene on exon 3 (c.130C>T; p.R44X) mutation	14% base editing, restored visual function, 89% editing in Rpe65 cDNA	[382]
April 2022	LCA 2	*Rpe65*	NG-ABE	Spit dual-AAV2 in rd12 mice, in vivo	Correct the *RPE65* mutation	Corrects up to 40% of Rpe65 transcripts, restores cone-mediated visual function, and preserves cones	[268]
February 2023	LCA2	*Rpe65*	PE3	Split dual-AAV8 in rd12 mice, in vivo	Correct the *Rpe65* gene on exon 3 (c.130C>T; p.R44X) mutation	Editing efficiency increased by 16%, restored RPE65 expression, rescued retinal and visual function	[269]
March 2023	RP	*Pde6b*	PE-SpRY	Split Npu intein dual-AAV delivery in *Pde6brd10* mice, in vivo	Correct the mutation in the *PDE6B* gene (c.1678C>T; p.R560C)	Achieved 76% prime editing and rescued visual function	[272]
June 2020	HL	*TMC1*	AID-BE3.9max	In vivo via dual AAV vectors, *in mice*	Base-editor-mediated correction of loss of function mutation	2.3% bulk genomic correction, 33% cDNA correction. Greatly increased hearing at 4 weeks that slowly degenerated	[157]
August 2024	HL	*OTOF*	ABE7.10max	Injected into the mouse inner ear using AAV, in vivo	Corrected the homozygous *OTOF* mutation (c.2482C>T, p.Q828X) in mice	The treatment restored otoferlin levels in 88% of inner hair cells, with stable effects lasting over 1.5 years	[285]
April 2018	DMD	*Dmd*	ABE7.10	Split dual trans-splicing AAV9 delivery in *Dmd* mice, in vivo	Base-editor-mediated correction of nonsense mutation	Restored dystrophin expression in myofibers in an adult DMD mouse model	[286]
April 2021	DMD	*Dmd*	ABE7.10max	Split-intein dual-AAV9 delivery in ΔEx51 mice, in vivo	Modify the splice donor sites of the dystrophin gene to cause skipping of exon 51 (Δx51)	35% local editing, 96% of local myofibers staining for restored dystrophin	[242]
	DMD	*Dmd*	PE3	Nucleofection in iPSC s, ex vivo	GT insertion in exon 52 of *DMD* (ΔEx51)	Successful restoration of myotonic protein expression	
January 2021	DMD	*Dmd*	twinPE	Transfection in HEK293 cells, ex vivo	Strategy for deleting exon 51 in *DMD*	Accurate deletions of up to 780 nucleotides were achieved with an efficiency of 28% and an indel rate of only 5.1%	[124]
July 2022	SMA	*SMN1/2*	PE3	Nucleofection in iPSCs*, *ex vivo	Targeted deletion of the splicing silencer N1 (ISS-N1) in *SMN2* to restore SMN expression	29% prime editing; FL-SMN expression was restored in the targeted-deletion iPS clones and their derived motor neurons	[247]
March 2023	SMA	*SMN1/2*	ABE8e	Split-intein dual-AAV9 delivery in *Δ7SMA* mice, in vivo	Exon 7 of the *SMN2* gene (C-to-T transition at position 6)	On average, 87% of SMN2 C6T was converted, restoring endogenous gene expression	[289]
January 2020	ALS	*SOD1*	BE3	In vivo via dual AAV vectors	Base-editor-mediated gene silencing	Improves neuromuscular function and slows disease progression	[156]
January 2020	NPC	*Npc1*	BE3.9max	Split-intein dual-AAV9 delivery in *Npc1I1061T* mice, in vivo	Base-editor-mediated correction of loss of function mutation	48% editing in cortex, 42% in Purkinje cells, up to 59% in cortex at test site. 10% increase in lifetime	[153]
May 2023	AD	*APOE3*	v3em PE3	Split-intein dual-AAV9 delivery in *APOE3* mice, in vivo	Introduce the putatively protective APOE Christchurch (APOE3 R136S) coding variant, a G-to-T transversion mutation	DNA editing efficiency in neocortex and hippocampus tissues was 12% and 14%, respectively	[118]
April 2024	AD	*MAPT*	NG-ABE8e	Trans-splicing AAV9 delivery in *PS19* mice, in vivo	Correct the *MAPT-P301S* mutant	The corrective efficiency was 5.7% ± 0.4%	[292]
June 2024	CF	*CFTR*	ABE8e	via the lung SORT LNP system to deliver to lung stem cells in mice	Correction of the *CFTR* nonsense mutation CFTR^R553X^	Over 50% of lung stem cell genes were successfully edited, with an overall lung cell editing rate of 12.2%	[298]
July 2024	CF	*CFTR*	PE6	Electroporation in primary airway epithelial, ex vivo	Correction of the *CFTR* F508del CTT deletion	Correction efficiency reached 58% in immortalized bronchial cells and 25% in patient-derived airway cells	[296]
September 2023	Cancer	*PTPRC*	ABE8e	Electroporation in CD45 + human cells, ex vivo	Inducing Point Mutations	Epitope-edited CD45 CAR T cells selectively target tumor cells without harming non-tumor cells	[305]
August 2023	Cancer	*FLT3; KIT; CD123*	SpRY-ABE8e	Electroporation in CD34 + human cells, ex vivo	Inducing Point Mutations	Epitope editing: mutating healthy tissue binding motifs to prevent off-tumor recognition by CARs/monoclonal antibodies	[306]
June 2023	Leukemia	CD52; CD7; TCRαβ	BE3	Electroporation in CD34+ human cells, ex vivo	Inducing Point Mutations	Base-Editing Donor T Cells to Target T-Cell Leukemia	[315]
March 2023	SCID	*CD3D*	ABE8e and ABE max	Electroporation in CD34^+^ CD3D-humanized mouse cells, ex vivo	Correct the c.202C>T mutation in the *CD3δ* gene	Edited HSPCs maintained 88% CD3D correction in humanized mice after 16 weeks	[301]
November 2023	CGD	*NCF1*	PE	Electroporation in CD34+ human cells*, *ex vivo	Correct the delGT mutation in the *NCF1* gene	Over 90% of CD34 cells were Prime Edited across 6 donors	[317]

Research by Mayuranathan and colleagues demonstrated that base editing has higher precision than Cas9 editing in treating sickle cell disease (SCD) because it does not rely on TP53-mediated DNA damage responses, thereby reducing side effects related to DNA repair pathways and avoiding unexpected phenotypic variations caused by insertions and deletions. Thus, base editing may be a superior option for treating SCD and other genetic disorders, offering a new direction for future gene therapies [233].

In early-stage examples of editing blood disorders, Zhang et al. used PE3 to edit the β-thalassemia IVS-II-654 mutation in mouse embryos, successfully correcting the *β-globin* gene splicing, achieving 14% editing efficiency, and eliminating thalassemia symptoms [234] (Fig. 6A). Another study using a non-integrating HDAd-PE5max vector for in vivo gene editing achieved promising results, with 40% β-globin S allele editing efficiency in an SCD mouse model, significantly alleviating SCD symptoms without observed off-target effects [235]. These results demonstrate the immense potential of the PE system in correcting genetic hematologic diseases and further validate the application prospects of gene editing technologies in precision medicine.

### Heart

**Fig. 5:**
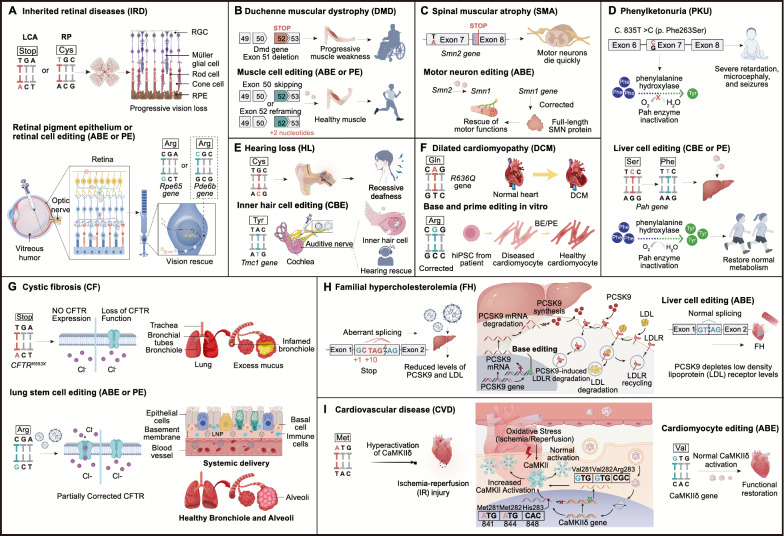
In vivo applications of precision genome editing technology for the treatment of human diseases. The current DNA base editing technology has been applied to disease models involving multiple organs, such as the eyes, ears, heart, liver, lungs, and muscles. Ophthalmic diseases include Leber congenital amaurosis (LCA) and retinitis pigmentosa (RP). Other diseases, such as liver diseases like phenylketonuria (PKU), muscle diseases like Duchenne muscular dystrophy (DMD) and spinal muscular atrophy (SMA), cardiovascular diseases such as familial hypercholesterolemia (FH) and dilated cardiomyopathy (DCM), and lung diseases like cystic fibrosis (CF), are also included. The figure illustrates the typical mutations and correction strategies for each corresponding disease

Liu and colleagues used the ABE to correct the Hutchinson-Gilford Progeria Syndrome (HGPS) mutation in a mouse model, achieving a correction rate of 20%–60%. This significantly improved the disease phenotype and extended the lifespan of the mice, demonstrating the potential of in vivo base editing for therapeutic applications [236, 237] (Table 3). Base editing has also shown significant effects in the treatment of hypertrophic cardiomyopathy (HCM). For example, using a dual AAV9-ABE8 system, over 70% editing efficiency was achieved in mouse cardiomyocytes, effectively preventing HCM-associated cardiac pathological changes. This effect lasted for 32 weeks, although 3%-5% bystander editing and a low but significant off-target editing rate were observed [80]. Additionally, the ABEmax-NG system corrected the *Myh6* gene mutation in HCM mouse embryos, achieving editing efficiencies of 62.5%–70.8%, reducing the expression of mutated RNA, and successfully rescuing the HCM phenotype in neonatal mice and their offspring [238]. Recent studies have further elucidated the potential of base editing in HCM treatment. For example, an AAV9-based split intein system has been shown to mitigate cardiac damage or disease by editing pathogenic variants in MYH7 [239], kinase IIδ (CaMKIIδ) [240] (Fig5I), and the pathogenic myosin variant R403Q [241] (Table 3).

Beyond directly correcting pathogenic mutations, base editing has emerged as a crucial tool for inducing exon skipping. A recent example involves the use of ABEmax to correct the ΔEx51 mutation, which causes Duchenne muscular dystrophy (DMD). This approach achieved a 35% local editing efficiency in muscle fibers and restored dystrophin expression in ΔEx51 mice [242]. Additionally, exon skipping successfully restored dystrophin levels in patient-specific hiPSC-derived cardiomyocytes, providing clinically relevant evidence for the prevention of DMD-associated dilated cardiomyopathy (DCM) [243, 244]. Notably, exon deletion mutations in the *DMD* gene are concentrated in specific regions, accounting for approximately 68% of all mutations, with exon 51 deletion being the second most common variant. This suggests that therapies targeting ΔEx51 could have significant clinical value for a substantial number of DMD patients [242] (Table 3).

Although base editing has shown high efficiency in correcting the p.R634Q mutation in the *RBM20* gene associated with dilated cardiomyopathy (DCM) [245], its application is limited by narrow activity windows, undesired edits, and the absence of suitable PAM sequences near certain target nucleotides. To overcome these limitations, Nishiyama et al. [245] utilized PE3b max in combination with epegRNA to successfully correct the DCM-associated p.R636S (c.1906C>A) mutation in a hiPSC cell line, achieving a 40% correction rate of mutant transcripts with no detectable mutations at any potential off-target sites, as confirmed by deep sequencing [245] (Fig. 5F). In another study, Zhang and his team employed dual AAV delivery of the PAM-less adenine base editor SpRY-ABE8e, which they developed, to effectively and precisely correct the *Mybpc3* gene mutation (Mybpc3c.2836C>T) in a hypertrophic cardiomyopathy (HCM) mouse model. This intervention prevented cardiac hypertrophy and dysfunction in the mouse model. Notably, this therapeutic strategy exhibited cardiomyocyte specificity and demonstrated low levels of bystander editing activity [246] (Table 3).

Prime editing is a highly promising and efficient tool with broad prospects for treating cardiovascular diseases. Compared to other gene-editing technologies, PE offers the advantage of reduced off-target effects, making it an ideal therapeutic option. In the field of cardiac research, prime editing has demonstrated high efficiency in generating Duchenne muscular dystrophy (DMD) cell and animal models, which may eventually contribute to the discovery of new drug targets or potential therapies [242, 247, 248]. However, the in vivo application of prime editing is currently limited by the challenges associated with delivering it to specific tissues or organs [113, 114, 151]. Therefore, significant barriers to efficiently editing the genomes of cardiac cells in vivo must be overcome to bring this technology closer to clinical application.

Increasing evidence suggests that many human diseases, such as coronary artery disease, are polygenic [249]. However, most existing genome-editing tools face limitations in precise multiplex editing, making it difficult to address multiple DNA editing needs simultaneously. Prime editing, on the other hand, can utilize tandem pegRNA arrays to achieve various required edits [140, 250, 251]. A single pegRNA or paired pegRNAs can be employed to correct multiple distinct mutations within a gene mutation hotspot. Thus, the same PE therapeutic approach has the potential to treat heterogeneous patient populations with different gene mutations.

### Liver

#### Familial hypercholesterolemia

PCSK9 is closely associated with cardiovascular disease, as the protein it encodes influences blood cholesterol levels by regulating the number of low-density lipoprotein (LDL) receptors on the surface of hepatocytes (Fig. 5H). Patients with hypercholesterolemia exhibit reduced LDLR expression, which can lead to elevated serum LDL-C levels and subsequent heart disease. Since PCSK9 is a negative regulator of LDLR, its inactivation can increase LDLR expression, thereby restoring LDL-C levels to a healthy range [243, 252, 253]. In animal studies [57, 58, 144, 163, 165, 254, 255], targeting PCSK9 has been identified as a favorable strategy because it offers a one-time, permanent, and precise therapeutic approach that may avoid the side effects associated with current oral LDL inhibitors, such as statins [256]. These studies lay the groundwork for developing BE/PE-mediated clinical trials (Table 3).

#### Phenylketonuria (PKU)

Phenylketonuria (PKU) is caused by mutations in the phenylalanine hydroxylase (PAH) gene, leading to the accumulation of phenylalanine in the body. Current dietary and pharmacological treatments cannot fully restore normal levels of phenylalanine in the blood [59]. Gene editing technologies, especially base editing and prime editing, offer a promising treatment option that can safely and permanently restore phenylalanine levels with a one-time intervention.

Studies have shown that base editors have demonstrated significant effects in correcting pathogenic mutations in the livers of PKU mice. They successfully restored blood phenylalanine levels to normal and rescued associated weight loss phenotypes [152, 257, 258] (Fig. 5D). Compared to other delivery methods, the lipid nanoparticle (LNP) delivery system appears to have a clear advantage in gene editing therapy, achieving complete correction and sustaining normal blood phenylalanine levels within 48 h of treatment [258](Table 3).

Prime editing, with its technical advantages, has also been quickly applied to the treatment of PKU, yielding positive results. For instance, using PE3 to edit the Pahenu2 mouse model carrying the homozygous mutation (c.835T>C; p.F263S), researchers achieved an editing efficiency of 11.1%, significantly reducing blood phenylalanine levels, with effects lasting for 18 weeks [113]. In another study, PEmax technology corrected the c.1222C>T (p. Arg408Trp) mutation in humanized PKU mice, achieving a correction efficiency of 52%, and restoring normal blood phenylalanine levels [259]. These results provide strong support for the potential of prime editing in PKU therapy (Table 3).

#### Hereditary tyrosinaemia type 1 (HT1)

HT1 is a rare genetic disorder caused by a deficiency in fumarylacetoacetate hydrolase (FAH), leading to the accumulation of toxic metabolites and subsequent liver dysfunction. Without timely treatment, patients may develop acute liver failure, cirrhosis, or even hepatocellular carcinoma. Historically, treatment has relied on strict low-protein diets and liver transplantation, but outcomes remain poor, driving researchers to explore new approaches, including gene therapy [260]. In several classical non-primate animal models, the prime editing (PE) system has shown promising therapeutic effects. Mice that received treatment exhibited significantly extended survival, reduced weight loss, and marked improvement in disease-associated phenotypes [96, 125, 137]. Furthermore, Jiang and colleagues successfully utilized a novel PEDAR system to delete a 1.38 kb pathogenic insertion fragment in vivo and simultaneously inserted a 19 bp DNA sequence at the target site, thereby restoring FAH expression in the livers of treated mice [125]. Notably, the prime editing (PE) strategy has also been successfully applied in ex vivo experiments. Kim et al. used electroporation to edit chemically derived hepatic progenitor cells (CdHs) from Fah^mut/mut^ mice and transplanted these cells back into the mouse liver. The PE-modified CdHs showed no significant off-target effects and successfully proliferated Fah-positive cells in the liver, significantly extending the survival time of the mice [261] (Fig. 4D). This demonstrates the feasibility of performing precise gene editing in transplantable cell populations for the treatment of genetic liver diseases.

#### α1-antitrypsin deficiency (AATD)

Genome editing technologies have brought hope to another severe genetic liver disorder—alpha-1 antitrypsin deficiency (AATD). AATD is caused by mutations in the Serpin Family A Member 1 (*SERPINA1*) gene, leading to the misfolding of alpha-1 antitrypsin, which accumulates in hepatocytes and induces endoplasmic reticulum stress, while the circulating levels of alpha-1 antitrypsin decrease, ultimately resulting in liver disease. Currently, aside from liver transplantation in cases of liver failure, there are few effective treatment strategies available [262].

One study demonstrated that low-dose split PE treatment was performed on *PiZ* mice (carrying the SERPINA1 E342K mutation) via tail vein injection of dual AAV8-PE3, achieving an editing efficiency of 3.1% ± 0.6% after 10 weeks with minimal indel events [114]. Another study also focused on this human disease. Notably, PE3 corrected the mutation in induced pluripotent stem cells (iPSCs) derived from AATD patients, with a gene correction frequency of 0.83%, which is comparable to that of the base editing (BE) system. This lower efficiency might be attributed to low transfection efficiency or the lower editing activity of the pegRNA used [263] (Table 3).

### Eye

Retinal diseases are attractive targets for gene therapy due to the accessible anatomical structure and the relatively low risk of immune responses [264]. Gene editing technologies offer revolutionary potential for treating inherited eye disorders such as Leber congenital amaurosis (LCA), retinitis pigmentosa (RP), and certain types of hereditary blindness [265] (Fig. 5A). Numerous preclinical studies and clinical trials have been conducted to explore treatments for these conditions. Below are some examples of gene editing approaches applied to ophthalmic diseases, each demonstrating unique advantages and limitations.

#### Leber congenital amaurosis (LCA)

Gene editing technologies have achieved significant results in repairing the *Rpe65* gene mutation associated with the hereditary retinal disease Leber congenital amaurosis type 2 (LCA2). The Palczewski team used a lentiviral vector with ABEmax, achieving a 15% editing rate, which restored retinal isomerase activity and improved visual function in mice [266]. Subsequently, the teams of Kim and Bae employed a dual AAV vector carrying an ABEmax variant to treat LCA2 mice, achieving editing rates of 10%-14%. Further sequencing showed that the editing efficiency in Rpe65-expressing cells reached as high as 80% [267] (Table 3).

Optimized base editor variants have further improved gene repair efficiency. For example, the enhanced split ABE (NG-ABE) dual AAV2 strategy achieved a 40% mRNA repair efficiency in rd12 mice [268]. The trans-splicing AAV2-SpCas9-NG-PE2 strategy repaired the *Rpe65* gene point mutation with a 28% efficiency, with no significant off-target effects detected [137]. Additionally, the dual AAV8 split PE3 technology restored *Rpe65* gene expression with a 16% efficiency and improved visual function in mice [269].

To improve delivery safety, non-viral delivery methods are also being explored. For example, liposomal delivery of base editors and gRNA ribonucleoprotein complexes achieved a 5.7% repair efficiency in RD12 mice [270]. These studies demonstrate the potential of base editing technologies in treating LCA2 and offer new directions for further optimizing delivery systems.

#### Retinitis pigmentosa (RP)

Retinitis pigmentosa (RP) is a progressive retinal degenerative disease that poses a serious threat to global visual health. Studies have shown that the R560C mutation in phosphodiesterase 6b (*PDE6b*) is closely associated with this hereditary retinal disorder [271]. Qin and colleagues [272] developed a novel gene editing tool named PE^SpRY^, characterized by SpRY unrestricted PAM requirements. This system, along with paired gRNAs, was delivered into rd10 mice—a well-established model of RP—using a dual AAV system. Unlike age-matched controls that experienced complete blindness, the gene editing induced by the PE^SpRY^ system effectively protected photoreceptors and restored PDE6 phosphodiesterase activity (Fig. 5A). More notably, the visual function of the rd10 mice was significantly improved, a result that was thoroughly validated through detailed electroretinography (ERG) and behavioral assessments.

It is worth noting that most mutations causing RP and other inherited retinal diseases (IRD) are associated with the function and survival of photoreceptor cells [271, 273–278]. Therefore, given the complexity of neural structures, the direct utility of genome editing in retinal cells, especially for unhealthy or dying photoreceptors, will provide more compelling evidence for the potential application of these tools in the treatment of RP [279].

### Ear

Hereditary hearing loss (HL) is an auditory disorder caused by gene mutations or abnormalities. In addition to conventional treatments, such as hearing aids and cochlear implants, recent evidence suggests that gene editing methods based on programmable CRISPR–Cas systems can enable targeted genomic engineering for gene therapy in HL [280, 281]. However, these methods typically struggle to address mutations causing recessive loss-of-function, which is a key challenge in treating recessive hereditary HL. Recessive hereditary HL requires the correction, rather than disruption or silencing, of pathogenic alleles to prevent hearing loss, presenting a range of difficulties for gene therapy in HL.

Fortunately, the advent of base editing and prime editing technologies has brought breakthrough advances in HL treatment. In 2017, researchers first evaluated the delivery of lipid nanoparticle (LNP)-encapsulated base editors to the inner ears of mice [133]. The gene editing efficiency reached 1% to 2% in the stria vascularis, around 1% in the cochlear axis, and 0.3% to 0.6% in the organ of Corti. Subsequently, researchers used similar methods to edit the β-catenin gene in the inner ear, successfully regulating the Wnt signaling pathway to induce the proliferation of supporting cells, which then differentiated into hair cells, opening new directions for gene therapy in inner ear-related diseases [282].

The recessive mutations in the *TMC1* gene cause rapid degeneration of hair cells, leading to complete deafness. TMC1 mutations account for 4% to 8% of hereditary hearing loss in certain populations [283]. AAV-based gene therapy for recessive Tmc1 mutations in mice can partially restore hearing, but the effects are limited and do not genuinely edit or repair the recessive gene mutation [284]. David Liu and colleagues applied base editing technology to successfully repair a missense mutation in the Tmc1 gene in the inner ears of Baringo mice [157] (Fig. 5E), achieving a 50% editing efficiency and significantly improving the hearing of treated mice at 4 weeks post-treatment. This achievement represents the first successful application of base editors in treating recessive hearing loss. However, the therapeutic effect only lasted for about 4 weeks, highlighting the need for optimization of efficiency and durability in clinical translation.

Additionally, for congenital hearing loss primarily caused by mutations in the *OTOF* gene, researchers used AAV-packaged ABE7.10max to repair the Q829X mutation in the *OTO* gene. Successful gene repair was achieved in a mouse model, with no significant off-target effects observed. After treatment, the cochlear protein levels in inner hair cells were restored to 88%, and the hearing function of the mice remained stable at near wild-type levels for over 1.5 years [285]. So far, this is the longest observed effective result in the field of gene therapy for deafness in animal models.

### Muscular

Base editing and prime editing have shown effective and precise repair of Duchenne muscular dystrophy (DMD) gene mutations in mouse models, leading to the restoration of dystrophin expression and subsequent improvement in symptoms. These results highlight the potential of these technologies as therapeutic approaches for DMD.

In a study by Kim and colleagues, base editing was applied for the first time in skeletal muscles of DMD (DMD) mouse models. Using a dual AAV system to deliver split ABE, they successfully corrected a nonsense mutation in the *DMD* gene. The study achieved a 3.3% editing efficiency, restoring dystrophin expression in 17% of muscle fibers, with no observed bystander effects or off-target editing [286] (Fig. 5B). Although the AAV dose used was higher than what would be required for human patients, this achievement laid the groundwork for future clinical applications. Other studies have also employed prime editing (PE) systems to correct the c.8713C>T point mutation in the *DMD* gene, achieving around 22% editing efficiency in muscle-derived cells from DMD patients [287].

In addition to correcting point mutations in the *DMD* gene, base editors and prime editors can also restore dystrophin expression in DMD by inducing exon skipping. Based on this strategy, Chemello and colleagues used a dual AAV9 approach to deliver ABEmax and successfully disrupted the splice donor site of exon 50. This restored the correct reading frame in a mouse model with exon 51 deletion (ΔEx51 mice), resulting in 96% of muscle fibers expressing dystrophin [242]. It is important to note that disrupting splice sites can potentially activate atypical splice sites in that region, which might lead to unintended consequences [288]. Additionally, the study reported the use of the PE3 strategy, where prime editing was used to precisely insert nucleotides and reconstruct the exon 51 deletion mutation in cell models, restoring dystrophin expression and improving contraction defects [242] (Fig. 5B).

In another study, Anzalone and colleagues employed the twinPE method to successfully remove a 780 bp sequence from exon 51 of the *DMD* gene. This achieved a 28% editing efficiency, with 5% targeted insertion or deletion, and no off-target damage observed. This approach shows promise as a candidate technology for developing in vivo editing therapies [124]. These innovative therapeutic strategies expand the range of genomic editing technologies available for the treatment of DMD.

#### Spinal muscular atrophy (SMA)

Spinal muscular atrophy (SMA) is a neurodegenerative disease caused by mutations in the *SMN1* gene, leading to motor neuron degeneration and muscle weakness [289]. Zolgensma® (NCT06019637) is a gene replacement therapy that delivers a full-length SMN cDNA via AAV vectors, but its long-term expression stability still needs to be validated. The *SMN2* gene has mutations in exon 7, leading to abnormal splicing of SMN protein. To address this, Arbab and colleagues developed the ABE8e strategy, using a dual AAV9 system to repair mutations in the Δ7SMA mouse model. This successfully converted the endogenous *SMN2* gene into Smn1, restoring SMN protein levels [289] (Fig. 5C). This approach improved motor function and extended the average lifespan of mice with the disease. Furthermore, this strategy showed synergy with the antisense oligonucleotide drug nusinersen, which regulates SMN2 splicing, leading to enhanced therapeutic outcomes [289, 290]. This integrated approach holds significant potential for future clinical applications in the treatment of SMA. In another study, Zhou and colleagues applied the PE3 strategy to delete the ISS-N1 region of SMN2 in iPSC models, restoring full-length SMN protein expression. The editing efficiency was 29%, with no off-target effects [247]. This is the first study to demonstrate the therapeutic potential of targeting and deleting ISS-N1 through prime editing to restore FL-SMN expression for SMA treatment (Table 3).

### Central nervous system

The central nervous system (CNS) is considered one of the most challenging targets for gene therapy due to its inherent resistance to large molecules and its ability to defend against bacterial invasions (a reason CRISPR was developed). Two in vivo base editing studies targeting the CNS in mice have been reported [153, 156], both employing dual AAV-mediated split-intron methods. The first study introduced a stop codon in the *SOD1* gene in an ALS mouse model, successfully delaying the disease progression. The transduction efficiency was approximately 6.5%, with a nonsense mutation detection rate of 1.2%. Compared to the control group, the treated mice had an 11% increase in average survival time and a 85% delay from disease onset to death [156] (Table 3). The second study corrected the Npc1I1061T mutation in a Niemann-Pick disease mouse model [153], using an optimized dual AAV9 CBE approach that significantly extended the mice's lifespan. The editing rates in cortical cells and enriched Purkinje cells were 48% and 42%, respectively [153]. This is particularly crucial for treating Niemann-Pick disease [291]. Overall, these studies open up new possibilities for treating neurodegenerative diseases and emphasize the importance of optimizing delivery vectors for effectively introducing targeted mutations into the therapeutic tissues.

#### Alzheimer’s disease (AD)

Gee and colleagues [292] used AAV9 to deliver the adenine base editor NG-ABE8e, successfully correcting the P301S mutation in the *Mapt* gene in the PS19 transgenic mouse model. They achieved a 5.7% editing rate in the hippocampus, with an observer editing rate of only 0.35%. This led to a significant reduction in pathogenic tau protein levels and improvements in cognitive function (Table 3). In another study, a research team used a split protein v3em PE3-AAV9 system to introduce the APOE3 R136S variant into a humanized APOE3 mouse model [118], achieving 12% editing efficiency and 5.0% targeted chimerism (Table 3). Despite the lower in vivo editing efficiency of this protective variant, its potential remains significant.

Overall, these studies provide new gene therapy strategies for treating tau-related neurodegenerative diseases and demonstrate the promising application of CRISPR base editing technology in treating complex diseases like Alzheimer's disease.

### Lung

Cystic fibrosis (CF) is an autosomal recessive genetic disorder that affects approximately 160,000 people worldwide [293]. In CF patients, mutations in the *CFTR* gene impair the function of its anion channel, which is responsible for transporting Cl^−^ and HCO_3_^−^ across the apical membranes of the pancreas, gastrointestinal tract, and airways [294, 295] (Fig. 5G). Among these mutations, the deletion of three base pairs (CTT) in the *CFTR* gene, known as F508del, is the leading cause of CF, with approximately 85% of patients carrying this mutation [296, 297]. Gene editing strategies offer a potential one-time therapeutic solution for correcting the CFTR F508del mutation. By utilizing a range of optimized strategies, including improved PE, guide RNA, PEmax architecture, and transient expression of a dominant-negative DNA mismatch repair inhibitor (MLH1dn), the efficiency of correcting the CFTR F508del mutation in patient-derived airway epithelial cells can be significantly enhanced [296]. Correction efficiency increased from less than 0.5% in immortalized bronchial epithelial cells to 58%, with 25% correction efficiency in patient airway epithelial cells. Compared to the Cas9 nuclease method, the new approach reduced unnecessary insertions and deletions per edit by 3.5-fold. This optimized PE restored CFTR ion channel function to over 50% of wild-type levels, comparable to the effect of current therapies such as Trikafta. Another study explored the possibility of achieving long-term gene correction in lung stem cells through in vivo gene editing technology [298]. The research team used an optimized lung-targeted lipid nanoparticle (lung SORT LNP) system to deliver the ABE8e gene editing tool to lung stem cells, successfully editing the *CFTR*^*R553X*^ nonsense mutation back to wild-type *CFTR*. In humanized CFTRR553X mutant mice, over 50% of lung stem cells were successfully edited, with an overall lung cell editing rate of 12.2%. Notably, compared to viral and other non-viral aerosol delivery methods, intravenously delivered SORT LNPs may more easily reach basal lung stem cells because they bypass the disease-related mucus barrier (Fig. 5G) and are closer in proximity to the lung endothelial bed [298, 299]. This study lays the groundwork for the development of long-term gene therapies for CF and other genetic lung diseases.

### Other diseases and conditions

#### Severe combined immunodeficiency, SCID

Severe combined immunodeficiency (SCID) caused by mutations in the *CD3δ* gene is a life-threatening congenital immune deficiency that results in a severe deficiency of T cells, typically leading to death during infancy. The traditional treatment is allogeneic hematopoietic stem cell transplantation (HSCT), which carries the risk of graft-versus-host disease (GvHD) and has a relatively low survival rate for patients [300]. To avoid GvHD, researchers have explored the potential of autologous transplantation using the patient’s own genetically modified hematopoietic stem cells. Gene editing based on CRISPR-Cas9 has been limited by the risks of off-target effects and low repair efficiency. Recent studies have shown that using the adenine base editor (ABE) strategy can correct approximately 71.2% of pathogenic mutations in hematopoietic stem cells from CD3δ SCID patients, and after transplantation into immunodeficient mice, about 88% of the mutations were corrected (Table 3), offering new hope for the treatment of CD3δ SCID and demonstrating its preclinical efficacy [301] (Fig. 4C).

#### Cancer

Precision and personalized therapy represent significant advancements in cancer treatment, particularly in the intervention of hematologic malignancies, where targeted immunotherapies such as ADCs, BTEs, and CAR T cells have achieved remarkable success [302–304]. However, CAR T-cell therapy for solid tumors faces substantial engineering challenges due to the lack of tumor-specific target antigens and the ongoing risk of on-target, off-tumor toxicity (OTOT). CD45, an antigen expressed on most hematopoietic cells and hematologic tumor cells, has the potential to serve as a universal antigen. Nevertheless, CAR T-cell therapy targeting CD45 would result in severe pancytopenia. To overcome this limitation, Wellhausen and colleagues developed a novel CAR T-cell therapy utilizing adenine base editors (ABE) to create an “epitope editing” strategy. This strategy involves inserting a nonsense mutation into the CD45 epitope, resulting in a single amino acid substitution in the targeted CD45 epitope. The modification allows anti-CD45 CAR T cells to avoid recognizing and targeting these edited cells while efficiently targeting blood cancer cells expressing normal CD45, including patient-derived acute myeloid leukemia, B-cell lymphoma, and acute T-cell leukemia. Importantly, these edited cells can still express CD45 and maintain their intracellular phosphatase function. Unlike directly knocking out CD45 in hematopoietic stem cells, epitope-edited hematopoietic stem cells can engraft, persist, and differentiate, thereby generating new blood cells [305] (Fig. 4B). Moreover, another study employed base editing to modify surface epitopes of transplanted hematopoietic stem and progenitor cells, eliminating CAR T-cell binding sites. This approach ensures that healthy transplanted cells are shielded from OTOT while malignant cells remain susceptible to CAR T-cell-mediated killing [306].

## Gene editing in clinical progress

Based on the programmability and powerful activity of CRISPR-based tools, they can be rapidly applied to the treatment of various indications, including in vitro cell engineering and in vivo gene correction prior to cell therapy. So far, the most common clinical CRISPR strategies have focused on genetic diseases of tissues that are more accessible to CRISPR nucleases, such as solid and liquid tumors, as well as liver, blood, and retinal diseases [225, 226, 307]**.** Thanks to the widespread use of CRISPR-Cas9-based gene editing tools in clinical trials, a solid foundation has been laid for the rapid development of base editors and prime editors [225]. However, traditional CRISPR methods work by cutting DNA to disrupt faulty genes, whereas base editors and prime editors offer more precise and safer functionalities. The growing pipeline of base editing and prime editing showcases the diverse potential of these therapies (Table 4). In this review, we highlight several recent clinical application cases, which are crucial for further understanding and unlocking the full therapeutic potential of gene editing technologies.

**Table 4:** Interventions based on base editing and prime editing are being developed

Disease indication	Therapeutic approach	Delivery strategies	Drug (sponsor)	status	Clinical trial
Heterozygous familial hyercolosterolemi (HeFH)	*PCSK9* silencing in liver	In vivo LNP	VERVE-101; VERVE-102 (Verve)	Phase 1b; Phase 1b	NCT05398029, NCT06164730
Familial hypercholesterolemia Atherosclerotic cardiovascular disease	Disrupting expression of *ANGPTL3* gene	In vivo LNP	VERVE-201 (Verve)	Phase 1b	NCT06451770
Sickle cell disease (SCD) β-thalassemia	Activation of fetal Hemoglobin	Ex vivo HSCs	BEAM-101 (Beam)	Phase 1/2	NCT05456880
Sickle cell disease	Correction of HbS sickle mutation	Ex vivo HSCs	BEAM-102 (Beam)	IND-enabling studies	N/a
β-thalassemia	Multiplex CD117 Edit-antibody pair	Ex vivo HSCs	ESCAPE (Beam)	Research/lead optimization	N/a
T cell ALL; CD7^+^ AML	Multiplexed silenced CD7 CAR-T	Ex vivo T cells	BEAM-201 (Beam)	Phase 1/2	NCT05885464
Glycogen storage disease 1a (GSD1a)	Correction of R83C mutation	In vivo LNP	BEAM-301 (Beam)	IND-enabling studies	N/a
α-1 Antitrypsin deficiency (AATD)	Correction of E342K mutation	In vivo LNP	BEAM-302 (Beam)	Phase 1/2	NCT06389877
Glycogen storage disease 1a	Correction of Q347X mutation	In vivo LNP	Unnamed candidate (Beam)	Research/lead optimization	N/a
Hepatitis B virus	Multiplex silencing	In vivo LNP	Unnamed candidate (Beam)	Research/lead optimization	N/a
Stargardt disease	Correction of G1961E mutation	In vivo AAV	Unnamed candidate (Beam)	Research/lead optimization	N/a
Relapsed/refractory T-cell ALL	Multiplexed silenced CD7 CAR-T	Ex vivo HSC’s	BE CAR7+ T cells/BECAR7 (GOSH)	Phase 1	NCT05397184
Chronic granulomatous disease (CGD)	Correct the delGT mutation in the NCF1 gene	Ex vivo HSC'	PM359 (Prime Medicine)	IND-enabling studies	NCT06559176

CASGEVY™ is the world’s first CRISPR clinical gene editing therapy to receive regulatory approval for the treatment of sickle cell disease (SCD) and transfusion-dependent β-thalassemia (TDT) [228], marking a breakthrough that ushers in a new era for CRISPR genome editing technologies.

**Fig. 6:**
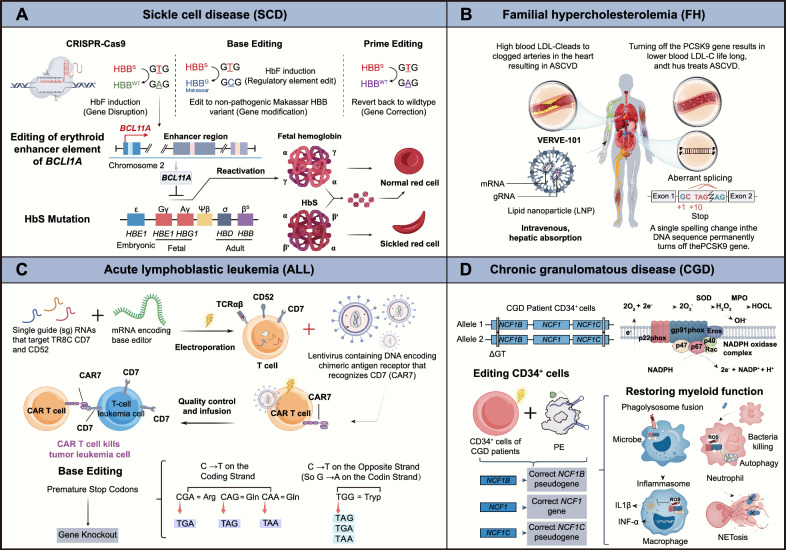
Clinical applications. **A** CRISPR-Cas9 can be used to correct the mutation back to the wild type or induce fetal hemoglobin (HbF) expression by altering the erythroid-specific enhancer BCL11A. Adenine base editors can edit regulatory elements controlling HbF expression or convert the HBB^S^ mutation to the non-pathogenic HBB^G^ Makassar variant. Prime editing can correct the HBB mutation to the wild type without inducing double-strand breaks. **B** A novel CRISPR base editing drug aims to inactivate PCSK9 in the liver by altering a single DNA base pair, thereby reducing LDL-C levels. **C** Base Editing Technology for Engineering Donor T Cells to Target T Cell Leukemia. Cytosine deamination via base editing allows for highly specific C → T conversions and can disrupt gene expression by introducing stop codons or removing splice sites without causing double-strand DNA breaks. BE-CAR7 T cells are generated by electroporating peripheral blood lymphocytes from a healthy donor with three sgRNAs targeting TRBC, CD7, and CD52, combined with mRNA encoding codon-optimized BE3. This process allows for CAR7 expression after lentiviral transduction without fratricide, enabling specific targeting of CD7^+^ leukemia cells. **D** Prime editing is used to correct the pathogenic mutations in the *NCF1* gene that cause chronic granulomatous disease (CGD). Correcting this gene or the pseudogene in cells from CGD patients can restore the expression of p47phox, thereby restoring the activity of NADPH oxidase

Casgevy™ originates from CTX001, an ex vivo CRISPR-Cas9 gene-editing therapy specifically designed for patients with TDT or severe SCD. It achieves this by editing hematopoietic stem cells (HSCs) to promote high levels of fetal hemoglobin (HbF) expression in red blood cells [308]. This process involves using Cas9 RNP electroporation to target and disrupt the erythroid enhancer of the *BCL11A* gene in CD34+ hematopoietic stem cells. The method achieves an 80% editing efficiency, significantly enhancing HbF production, effectively alleviating symptoms associated with TDT and SCD, such as vaso-occlusive crises (Fig. 6A). These results have been validated in multiple clinical trials (e.g., NCT03655678, NCT03745287). The experimental results will provide deeper insights into the comparison between these editors and other genome editing tools.

Significant progress has been made in clinical trials for the treatment of familial hypercholesterolemia (FH). The first base editing pipeline to enter clinical trials was developed by Verve Therapeutics, which uses lipid nanoparticles (LNPs) carrying sgRNA and an adenine base editor to treat heterozygous familial hypercholesterolemia (Fig. 6B). Genetic biology mechanisms [253, 309] and pharmacological treatments [243, 310, 311] have demonstrated that inhibiting PCSK9 can lower LDL levels. The study involves a drug known as VERVE-101, composed of mRNA encoding ABE and guide RNA, which are packaged within lipid nanoparticles and administered via intravenous infusion [57, 58, 312]. A particularly detailed preclinical study [312] evaluated liver enzymes, off-target editing, germline editing, and efficacy up to 476 days after a single dose, with the drug being administered to patients in New Zealand and the UK. The clinical trial is currently in the Phase 1b stage (Table 4). Interestingly, this project encountered a clinical hold from the FDA, which requested additional data, including therapeutic effects in both human and non-human cells, off-target delivery (i.e., non-liver cells), germline editing, and clinical data from the UK and New Zealand.

Verve has also developed VERVE-201, an LNP-based base editing therapy designed to inactivate the *ANGPTL3* gene [313]. Genetic and pharmacological inhibition experiments have demonstrated that this gene is another risk factor for heart disease [314]. In this study, *N*-acetylgalactosamine (GalNAc) ligands were added to bind with asialoglycoprotein receptors specifically expressed on hepatocytes, enhancing liver cell targeting. A single treatment resulted in a 96% reduction in circulating ANGPTL3 levels within 616 days. Currently, VERVE-201 has entered the Phase 1b stage and has not yet been administered to patients, but the company has initiated research on new drugs.

Currently, several clinical trials of CRISPR base-edited CAR-T cell therapies are underway, targeting a range of hematologic cancers. A notable example is the use of multi-base-edited CAR-T cells to treat relapsed T-cell leukemia in pediatric patients, a previously incurable condition [315] (Fig. 6C). Notably, on December 2, 2022, the FDA approved Beam Therapeutics’ Investigational New Drug (IND) application for BEAM-201, a base-edited CAR-T cell therapy for the treatment of relapsed/refractory acute T-cell lymphoblastic leukemia (T-ALL) and T-cell lymphoblastic lymphoma (T-LL). BEAM-201 therapy involves the base-editing knockout of four genes: *CD7*, *TRAC*, *CD52*, and *PD-1.* Compared to the base-edited CAR-T therapy published in NEJM, BEAM-201 includes an additional PD-1 knockout, aimed at enhancing the persistence of CAR-T cell antitumor activity. The base editing process is highly efficient, with an editing efficiency ranging from 96 to 99%. The BEAM-201 therapy is currently in the Phase 1/2 stage (Table 4). These genetically engineered CAR-T cells can serve as a “universal” cell therapy, meaning this therapy can become an “off-the-shelf” product available to many patients, in stark contrast to the currently time-consuming and costly personalized T-cell therapies. In addition to these therapies, clinical trials for other drugs aimed at silencing, repairing, regulating, or upregulating genes by altering specific single nucleotide sites in patients' genomes are also about to begin (Table 4). The success of early clinical trials of base editing therapies suggests that gene editing is applicable not only to rare diseases but also to common diseases. However, the extraordinary precision of this technology comes at the cost of flexibility: it can only be used to modify certain DNA sequences and cannot insert large DNA segments into the genome. In contrast, prime editors have the potential to overcome the functional limitations of current base editing, offering greater targeting flexibility and versatility, but are also more complex. As researchers continue to push the boundaries of this technology, clinical trials for prime editing are gradually becoming a reality.

On April 29, 2024, Prime Medicine announced that the Investigational New Drug (IND) application for its lead editing therapy targeting chronic granulomatous disease (CGD) had been approved by the U.S. Food and Drug Administration (FDA), paving the way for the initiation of a global Phase 1/2 clinical trial [316] (Table 4). Chronic granulomatous disease is caused by the ΔGT (75_76delGT) mutation in the *NCF1* gene, which prevents the successful translation of a functional protein. This mutation impairs the function of phagocytes by reducing the production of nicotinamide adenine dinucleotide phosphate (NADPH) and the ability of phagocytes to generate bactericidal reactive oxygen species (ROS) (Fig. 6D). In this context, correction of the *NCF1* gene itself or any processed pseudogene could rectify this disorder, thereby increasing the likelihood of achieving functional therapeutic benefits [317] (Fig. 6D).

## Clinical considerations and challenges in gene editing

### Considerations in therapeutic gene editing

The emergence of programmable genome editing technologies has paved the way for the application of cell and gene therapies in the treatment, and even potential cure, of diseases. It is important to note that before genome editing can be widely applied in human patients, several challenges must be addressed. The clinical efficacy of genome editing fundamentally depends on its specificity and precision. To achieve effective editing, sufficient delivery systems are required to ensure adequate genetic modification in the targeted cells. This depends not only on the intrinsic activity of the gene editors but also on the methods used to deliver them to the target cell types and tissues. In particular, the safe, specific, and efficient delivery of CRISPR components to target cells is a prerequisite for the successful therapeutic genome editing (see “Methods for delivering genome-editing tools” section for further details).

The specificity of genome editing describes the ratio of targeted to off-target genetic changes, some of which can be predicted and tested using various methods [318]. Associated with editing efficiency is precision, which enumerates how many modifications are perfect and how many are undesired. The precision of base editors (BEs) makes them suitable for treating diseases caused by single-base mutations. Although BEs can more precisely control editing outcomes, they also have certain limitations. These limitations include limited efficiency, bystander editing, wide editing windows, and significant off-target activity [319–321]. Furthermore, both adenine base editors (ABE) and cytosine base editors (CBE) may cause undesirable genomic toxic side effects by generating double-strand breaks (DSBs), deletions, and translocations at the target loci, although the frequency is lower than that of typical nuclease-based Cas9 editing. These issues can currently be partially mitigated by adjusting delivery timing and the expression levels of the editors [319]. For prime editing, editing efficiency typically varies greatly and remains low depending on the expected edits, target site sequence, and cell type [322]. Additionally, prime editing does not entirely avoid the creation of target DSBs, potentially leading to unintended and possibly toxic genetic effects [319]. These limitations of prime editing present opportunities for further development, such as the creation of new prime editing variants with better performance [96, 115, 323], including systems that use pegRNA to achieve more extensive editing [124, 126–128, 324].

Finally, another challenge facing this field is the identification of models for measuring efficiency, precision, and specificity. It can be difficult to comprehensively test genome modifications using experimental models in a therapeutic context, as non-human cells are expected to have different off-target sequences compared to human cells with a given genomic sequence. Therefore, experiments in non-human models may not fully replicate the in vivo conditions associated with therapeutic delivery. Moreover, due to physiological and genomic structural differences between mice and humans, mouse models often cannot fully replicate the key pathological changes and/or important symptoms of human diseases. Large animal models, on the other hand, can bridge the gap between small animal research and clinical trials [325–327]. As a result, preclinical research data, particularly studies in non-human primates and large animals, are critical steps in advancing the successful translation of gene editing technologies into human therapies [312, 328]. Additionally, closely monitoring clinical trial participants and conducting relevant molecular analyses may be an effective method for truly assessing the comprehensive outcomes of genome editing.

### Clinical risk considerations in gene editing

Currently, gene editing methods are still in the early stages of clinical development, so their long-term safety and associated clinical risks warrant close attention. Although an increasing number of technologies can be used to classify target and off-target genomic effects [318], somatic gene modifications themselves may not directly lead to clinical consequences. Over time, as cells divide, they naturally accumulate DNA damage, so a one-time, modest increase in somatic genetic variation may not have a significant impact. Overall, most genetic variations are expected to be neutral, and the few variations that may be harmful to cells typically do not have a significant clinical impact. In contrast, the most concerning cases may be those leading to gain-of-function mutations, which can promote clonal expansion and tumor formation by activating oncogenes or inhibiting tumor suppressors. To date, no accidental cases of therapeutic gene editing leading to tumors have been reported in clinical studies, suggesting that the frequency of such effects may be very low.

It is important to note that clinical considerations should extend to the entire gene editing therapeutic process, including any associated risks related to cell delivery and host responses, which may represent more common clinical risks. For in vivo therapies, this may include adverse reactions of the host to gene transfer vectors (such as AAV, AdV, LV, etc.) or transposons, which could trigger stress responses such as immune reactions and DNA damage responses [329]. As base editing technologies mature and standardize, their widespread deployment in clinical settings is expected to expand. This expansion highlights the need for stringent ethical review of the technology's applications. Key ethical considerations include the long-term impact of genome editing on individuals and society, necessitating comprehensive risk assessments and ethical deliberations before implementation [330].

Finally, for these considerations to be meaningful, therapeutic options must be available to patients. This requires addressing the regulatory hurdles that may impede the development of gene therapies and their applicability to rare disease patients. After therapy approval, high costs and complexity may hinder patient accessibility and widespread use. In general, simpler and more cost-effective therapies are more likely to be accepted by patients.We again extend our sincerest thanks to you.

## The future: emerging technologies

### DNA polymerase editor

**Fig. 7:**
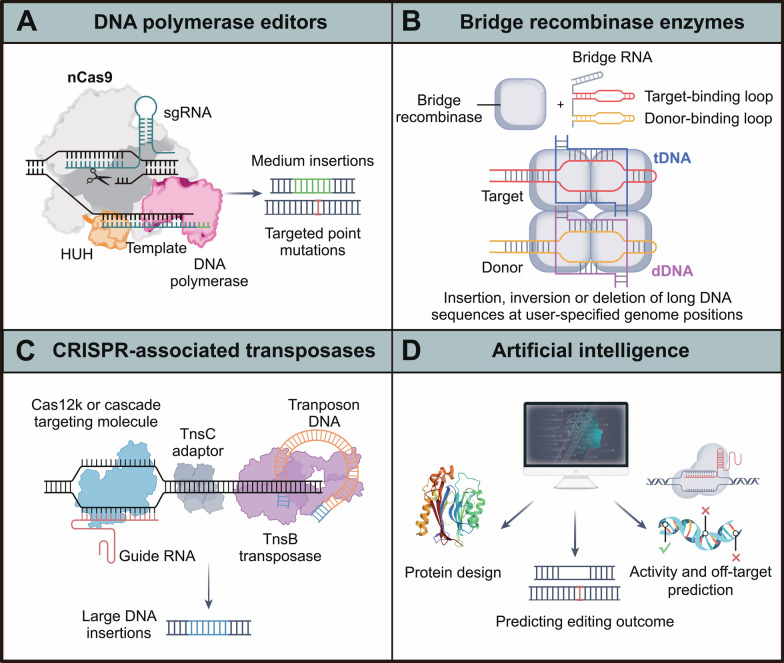
Summary of emerging technologies in genome editing. **A** This technology combines HUH nucleases (HUHe), DNA-dependent DNA polymerase (DDP), and RNA-guided Cas9 nickase (nCas9), enabling programmable and precise genome engineering from single-stranded DNA templates, including genome replacements, insertions, and deletions. A major distinction from prime editing is that it uses DNA polymerase instead of reverse transcriptase and delivers the DNA template in trans. **B** A recombinase guided by bridge RNA (Bridge RNA) can insert, invert, or delete long DNA sequences at specific genomic sites. This RNA contains two loop structures that bind separately to the donor and target sequences, as shown in the figure. These loop structures can be independently designed, enabling the recombinase to modify specified sequences. **C** A complex combining the CRISPR-Cas effector system with Tn7 family transposons not only retains some of the functions of the CRISPR-Cas system but also possesses the mobility of transposons. This enables RNA-guided transposition, allowing the insertion of long DNA sequences into specific genomic sites. **D** By predicting off-target effects and editing outcomes, artificial intelligence is poised to significantly enhance current gene editing technologies, particularly in the design of novel proteins and guide RNAs, where it has already achieved breakthrough advancements. These improvements can greatly increase the precision and efficiency of gene editing, laying a solid foundation for precise and safe gene therapies

Unlike prime editing, new methods are exploring the use of DNA polymerases to introduce specific mutations into the genome. For instance, by fusing engineered error-prone DNA polymerases with Cas9 endonuclease, nucleotide diversity can be achieved within a range of up to 350 nucleotides around the target site [331]. This approach facilitates the introduction of extensive genetic variation within a specific region, which is useful for functional gene screening and evolutionary studies. Another study demonstrated the use of phage-derived DNA polymerases to introduce edits at Cas9 cut sites using a linear DNA template, avoiding the issue of guide RNA self-inhibition and enabling the insertion of sequences over 100 nucleotides long [332]. This method not only enhances the precision of editing but also increases the potential for inserting long DNA fragments into the genome. Furthermore, a novel technique called “click editing” combines HUH endonucleases (HUHe), DNA-dependent DNA polymerases (DDP), and nCas9 to perform programmable, precise genomic engineering, including replacements, insertions, and deletions, from simple DNA templates [333] (Fig. 7A). This method does not require double-strand breaks (DSBs), thereby promoting accurate genome editing with minimal insertions and deletions, while avoiding unintended insertions. The strength of this method lies in its ability to achieve highly controlled gene editing without compromising genome integrity. DNA polymerase-based editing techniques offer substantial potential due to their capacity to induce various genetic changes. They provide more diverse and flexible editing outcomes compared to traditional gene editing techniques, allowing scientists to precisely manipulate the genome, thereby advancing gene function research, disease modeling, and potential therapeutic applications.

### Minimised ancestral RNA-guided nuclease

The exploration of novel RNA-guided nucleases aims to discover alternative enzymes with diverse guide RNA scaffolds, targeting capabilities, and smaller sizes to facilitate the construction of fusion proteins, such as base editors and prime editors, and to enable efficient cellular delivery. The recently developed ultracompact Cas12f nuclease (422 amino acids) exemplifies this pursuit [334]. These investigations primarily focus on the IscB/IsrB [335] and TnpB [336] proteins. Collectively referred to as OMEGA [337] proteins, these are encoded within prokaryotic transposable elements, contributing to transposition mechanisms and promoting the retention of transposons. Simultaneously, they possess the potential for programmable genome editing in human cells [335, 336]. Recently discovered eukaryote-like TnpB proteins, termed Fanzors [338–341] (approximately 400–700 amino acids), also catalyze RNA-guided DNA cleavage and support programmable genome editing [341], further demonstrating the ubiquity of RNA-guided nucleases across various life forms. Despite the compact size of these nucleases, which facilitates delivery, further molecular engineering is necessary to enhance their efficiency and targeting range.

### Recombinases and transposons

Template-guided double-strand DNA break repair, such as homologous recombination (HDR), is highly effective in proliferating cells but has limited success in post-mitotic cells [342]. As the size of the insertion fragment increases, the efficiency of HDR-mediated insertion progressively decreases, restricting the insertion of long DNA fragments. Although prime editing (PE) does not rely on HDR, it currently allows the insertion of only a few dozen nucleotides [84]. The integration capacity of PE has been extended by combining it with serine recombinases or integrases, enabling the insertion of DNA sequences up to several kilobases [123, 124, 323] (Fig. 2F). The discovery and optimization of novel recombinases will further enhance the efficiency and specificity of this approach [129, 343]. Transposons can autonomously insert large DNA segments without requiring double-strand breaks. Although Cas9-transposase fusion technology has increased transposition events near target sites, its low efficiency and high off-target frequency have limited its widespread application [344, 345]. In contrast, CRISPR-associated transposon (CAST) systems, which utilize type I or type V CRISPR-Cas systems, achieve RNA-guided DNA transposition and have demonstrated efficient and site-specific insertion in bacteria [346–348]. Extensive research has elucidated the mechanisms of CAST, laying the groundwork for its application in mammalian cells [349–353] (Fig. 7C). Despite the lower efficiency of type V CAST, type I CAST has shown potential in human cells, offering promise for the precise insertion of large genetic payloads. However, robust genome editing and therapeutic gene delivery will require further research and optimization [354, 355].

In-depth research on recombinases and transposases that mediate large-scale genomic rearrangements is instrumental in developing a programmable approach to rearrange long DNA sequences within the genome. Two recent papers [356, 357] reported on the characteristics of recombinases guided by “bridging” RNA molecules, which can be reprogrammed to unlock new genome editing capabilities. This research [356] demonstrated that IS110 encodes a recombinase and a non-coding bridge RNA, which can specifically bind to the encoded recombinase. The bridge RNA contains two internal loop structures with nucleotide sequences that can pair with target DNA and donor DNA (i.e., the IS110 element itself) (Fig. 7B). Further studies have shown that the target-binding loop and donor-binding loop can be independently reprogrammed to direct specific sequence recombination between two DNA molecules [357]. This modularity enables the insertion of DNA into genomic target sites, as well as programmable DNA excision and inversion. Overall, the modular nature of bridge RNA allows for a generalized DNA rearrangement mechanism through sequence-specific insertion, inversion, or deletion. The IS110 bridge recombination system extends the diversity of nucleic acid-guided systems beyond CRISPR and RNAi, thereby introducing a novel genome editing technology.

### Artificial intelligence (AI)

The rapid adoption of AI in the field of gene editing has had a profound impact, significantly enhancing the precision and efficiency of editing processes. AI has notably improved the ability to predict off-target effects and minimize unintended genetic alterations, thereby greatly increasing the safety and efficacy of therapeutic applications [107, 358–360]. Looking ahead, AI is expected to further contribute to personalized gene editing strategies and facilitate the development of more efficient and specific nucleases through novel computational protein design [361–364] (Fig. 7D). However, AI approaches also face challenges, such as their reliance on high-quality training data and the “black box” nature of AI, which can make the interpretation and trustworthiness of predictions difficult. Despite these challenges, the potential of AI in gene editing is immense, but careful attention must be paid to addressing these issues.

## Conclusion

The ideal gene editing technology would be capable of converting target DNA into any desired sequence while minimizing unwanted genomic modifications or cellular disturbances. Developing efficient, versatile, highly pure, and specific gene editing tools has always been a core objective in life sciences. The continuous advancement of new techniques and methodologies aimed at improving the accuracy and precision of genome editing has fundamentally transformed our ability to manipulate genetic material with ease and precision. These ever-improving technologies benefit from metagenomic exploration, synthetic biology, and AI-supported molecular engineering. Future trends will continue to focus on developing more effective and safer delivery vectors, reducing off-target effects, and achieving precise control over editing outcomes. As gene therapy technologies progressively move towards clinical applications, the bottleneck in targeted disease treatment will no longer be the safety, efficacy, or precision of genome editors, but rather the final step of clinical delivery, particularly to specific tissues, organs, and cells outside the liver. The development of efficient delivery methods for novel vectors and the identification of safe editing targets will expand the range of diseases that base editing and prime editors can treat. The rise of artificial intelligence and other machine learning methods marks the beginning of an era of interdisciplinary integration, which will facilitate more accurate simulations of the genome editing environment, predictions of outcomes, and the design of more powerful genome editors, thereby accelerating the implementation of safe treatments. We hold strong confidence that the continued research and development in this field will yield substantial progress, ultimately resulting in tangible benefits for patients.

In the coming years, these technologies are likely to play a progressively significant role in treating genetic diseases. However, the impact of genome editing technology on clinical ethics cannot be overlooked and demands thorough consideration. It is crucial to strike a balance between potential benefits, potential risks, and ethical considerations. Therefore, ongoing dialogue between scientists, policymakers, and the public is vital for ensuring the safe and responsible use of these technologies.

In summary, gene editing technology not only drives research breakthroughs but also fundamentally changes human medicine. Its potential, however, extends beyond these areas, playing a critical role in addressing ecological challenges, agricultural transformation, and disease prevention and control. The future of gene editing technology is indeed promising. Our discussion is confined to targeted alterations of genomic DNA sequences using CRISPR-based tools, and and we recommend readers explore excellent reviews on other CRISPR applications, such as transcriptional regulation [5, 225], epigenetic modifications [225], RNA editing [365, 366], Mitochondrial Base Editing [367], and transposons [6, 366].

## Data Availability

Not applicable.

## Abbreviations

CRISPR: Clustered regularly interspaced short palindromic repeats
PAM: Protospacer adjacent motif
pre-crRNA: Precursor CRISPR RNA transcript
sgRNA: Single-guide RNA
DSBs: Double strand breaks
NHEJ: Non-homologous end joining
HDR: Homology-directed repair
MMR: Mismatch repair pathway
nCas9: Nickase Cas9
dCas9: Nuclease-deficient Cas9
CBEs: Cytidine base editors
ABEs: Adenine base editors
ssDNA: Single-stranded DNA
CGBEs: C·G-to-G·C base editors
STEME: Saturated targeted endogenous mutagenesis editor
SPACE: Synchronous Programmable Adenine and Cytosine Editor
A&C-BE: Base editor catalyzes both cytosine and adenine base conversions
Target-ACE: Base editors for simultaneous introduction of C-to-T and A-to-G mutations
AGBE: A dual deaminase-mediated base editor by fusing CGBE with ABE for creating a saturated mutant population with multiple editing patterns
AXBEs and ACBEs (X = arbitrary nucleotides): Adenine transversion editors enable precise, efficient A·T-to-C·G base editing
AYBEs: Enabling base editing of A-to-C or A-to-T
gCBE: Glycosylase-based Cytosine Base Editor (T-to-C or T-to-G)
gTBE: Glycosylase-based thymine base editor (C-to-G)
DAF-CBE: Enabling base editing of C-to-A or T-to-A
DAF-TBE: Enabling base editing of C-to-G or T-to-G
TSBE: Enabling base editing of T-to-G or T-to-C
gGBE: Enabling base editing of G-to-C/T
HOPE: Homologous 3ʹ extension mediated prime editor
PEDAR: PE-Cas9-based deletion and repair
GRAND: Genome editing by RTTs partially aligned to each other but non-homologous to target sequences within duo pegRNA
PETI: Prime editor nuclease-mediated translocation and inversion
SNVs: Single nucleotide variants
RT: Reverse transcriptase
pegRNA: Prime-editing guide RNA
PBS: Primer binding site
NMD: Nonsense-mediated decay
AAV: Adeno-associated viruses
LVs: Lentiviruses
AdV: Adenoviruses
LNPs: Lipid nanoparticles
VLPs: Virus-like particles
HTI: Hereditary tyrosinemia type I
SCD: Sickle cell disease
TDT: Transfusion-dependent beta-thalassemia
CGD: Chronic Granulomatous Disease
AD: Alzheimer’s Disease
RP: Retinitis pigmentosa
LCA2: Leber congenital amaurosis (LCA) 2
SMA: Spinal muscular atrophy
FH: Familial Hypercholesterolemia
HCM: Hypertrophic Cardiomyopathy
DMD: Duchenne Muscular Dystrophy
PKU: Phenylketonuria
ALS: Amyotrophic Lateral Sclerosis
NPC: Niemann-Pick disease type C
DCM: Dilated Cardiomyopathy
CF: Cystic Fibrosis
CVD: Cardiovascular Disease
T-ALL: T-Cell Acute Lymphoblastic Leukemia
T-LL: T-Cell Lymphoblastic Lymphoma
RH: Refractory Hyperlipidemia
AATD: Adult Patients With Alpha-1 Antitrypsin Deficiency
HSPCs: Haematopoietic stem or progenitor cells
HbF: Fetal hemoglobin
HUHe: HUH nucleases
DDP: DNA-dependent DNA polymerase
